# Treating ischemia via recruitment of antigen-specific T cells

**DOI:** 10.1126/sciadv.aav6313

**Published:** 2019-07-31

**Authors:** Brian J. Kwee, Bo Ri Seo, Alexander J. Najibi, Aileen W. Li, Ting-Yu Shih, Des White, David J. Mooney

**Affiliations:** 1 John A. Paulson School of Engineering and Applied Sciences, Harvard University, Cambridge, MA 02138, USA.; 2 Wyss Institute Biologically Inspired Engineering, Harvard University, Boston, MA 02115, USA.

## Abstract

Ischemic diseases are a leading cause of mortality and can result in autoamputation of lower limbs. We explored the hypothesis that implantation of an antigen-releasing scaffold, in animals previously vaccinated with the same antigen, can concentrate T_H_2 T cells and enhance vascularization of ischemic tissue. This approach may be clinically relevant, as all persons receiving childhood vaccines recommended by the Centers for Disease Control and Prevention have vaccines that contain aluminum, a T_H_2 adjuvant. To test the hypothesis, mice with hindlimb ischemia, previously vaccinated with ovalbumin (OVA) and aluminum, received OVA-releasing scaffolds. Vaccinated mice receiving OVA-releasing scaffolds locally concentrated antigen-specific T_H_2 T cells in the surrounding ischemic tissue. This resulted in local angiogenesis, increased perfusion in ischemic limbs, and reduced necrosis and enhanced regenerating myofibers in the muscle. These findings support the premise that antigen depots may provide a treatment for ischemic diseases in patients previously vaccinated with aluminum-containing adjuvants.

## Teaser

Recruitment of antigen-specific T cells promotes vascularization in ischemic injuries in mice.

## INTRODUCTION

There exists a critical need to enhance vascularization in ischemic diseases, as these are a leading cause of mortality and morbidity worldwide. Peripheral arterial disease, in particular, results from thrombosis of arteries in the lower extremities, which can result in severe conditions such as ulceration, gangrene, and even limb amputation (*1*). There has long been an interest in treating these diseases with strategies that promote angiogenesis, the growth of new blood vessels from preexisting blood vessels, to bypass thrombotic clots (*2*). These strategies typically involve the systemic delivery of recombinant, angiogenic growth factors, such as vascular endothelial growth factor (VEGF) and basic fibroblast growth factor (bFGF), to directly stimulate new blood vessel formation (*3*). However, to date, large therapeutic angiogenesis clinical trials have failed, likely due, in part, to the short half-life and poor retention of delivered factors at the disease site and the need for multiple agents to support the induced vasculature (*4*). Biomaterials have overcome some of these shortcomings by providing local, controlled, and sustained release of angiogenic factors, such as VEGF, at sites of injury (*5*, *6*). However, the ability of these drug delivery biomaterials to enhance blood perfusion in vivo is greatly dependent on the presence and activity of immune cells in the host (*7*).

The important role of the immune system in angiogenesis and vascular remodeling has motivated efforts to promote revascularization in ischemic tissues via manipulation of immune cells. Monocytes have been shown to promote the remodeling of both preexisting and newly formed blood vessels (*8*, *9*). Furthermore, macrophages are known to differentially contribute to vascularization based on their phenotype, with M1 macrophages promoting the initiation of angiogenesis and M2 macrophages enhancing the maturation and remodeling of blood vessels (*10*, *11*). To date, immunotherapies for vascularization have primarily focused on using these cells of the innate immune system (*12*, *13*). For example, delivery of FTY20, an S1P_1/3_ agonist, from polymer films promoted arteriogenesis by enhancing the recruitment of anti-inflammatory monocytes (*14*). Controlled delivery of interferon-γ (IFN-γ) enhanced vascularization in subcutaneous tissue, presumably by altering the presence and polarization of M1 macrophages (*15*).

It is increasingly appreciated that CD4^+^ helper T cells, immune cells of the adaptive immune system, can also promote vascularization. These cells promote arteriogenesis in models of hindlimb ischemia (*16*, *17*) and help to reduce ischemic necrosis (*16*, *17*). Furthermore, T cells have been shown to be potent sources of a number of angiogenic factors, including VEGF, bFGF, and HB-EGF (heparin-binding epidermal growth factor-like growth factor) (*18*, *19*). T helper 2 (T_H_2) T cells, in particular, have recently been shown to secrete factors that are conducive for vascularization in ischemic injuries (*20*). In addition, T_H_2 T cells have been shown to activate eosinophils, which are also potent sources of angiogenic factors such as VEGF (*21*).

This study explored the ability of a biomaterial that concentrates antigen-specific T_H_2 memory T cells, already formed with previous vaccinations, to promote blood perfusion recovery of ischemic tissue. Virtually all persons born in the United States have received childhood vaccinations recommended by the Centers for Disease Control and Prevention, such as those for DTaP (diphtheria, tetanus, and pertussis) and hepatitis B, that contain aluminum, a potent T_H_2 adjuvant (*22*, *23*). A biomaterial that provides localized release of antigen specific to these T_H_2 memory T cells at later times in these patients may transiently recruit and activate these cells to produce cytokines at sites of ischemia and promote vascularization. To test this hypothesis, mice were first vaccinated using the model antigen ovalbumin (OVA) in the presence of aluminum (as a mimic for childhood vaccines) and later received an implantation of OVA-containing scaffolds in their ischemic hindlimbs. This vascularization strategy relies on (i) the widely accepted ability of aluminum as an adjuvant to generate memory T_H_2 T cells when used in combination with an antigen (*23*) and (ii) our previous work demonstrating that antigen-containing polymer scaffolds can enrich for antigen-specific T cells to the scaffold site (*24*).

## RESULTS

### Scaffolds concentrate antigen-specific T cells in ischemic muscle

**Fig. 1:**
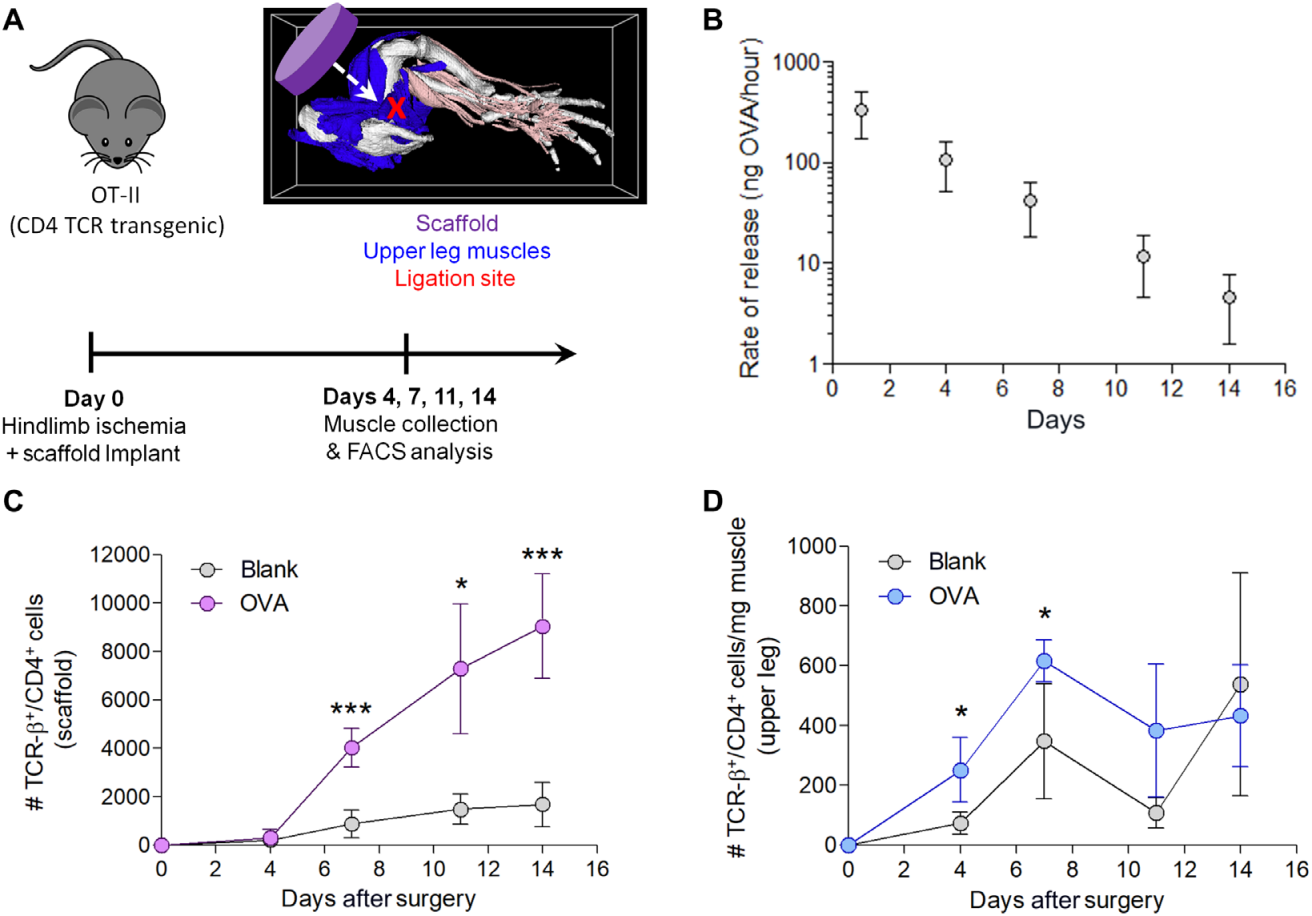
OVA-releasing scaffolds concentrate OVA-specific CD4^+^T cells following ischemic injury. (**A**) Experimental design using (upper left) OT-II mice (TCR transgenic mice where all CD4^+^ T cells are OVA specific), (upper right) diagram of scaffold implantation (purple) relative to ischemic upper leg muscles (blue) and ischemic ligation site (red), and (bottom) timeline of experimental procedures. White arrow indicates location of placement of scaffold in the upper right diagram. FACS, fluorescence-activated cell sorting. (**B**) Instantaneous rate of release in vitro of OVA from PLG scaffolds up to 14 days (*n* = 3 scaffolds). (**C** and **D**) Number of CD4^+^ T cells (TCR-β^+^/CD4^+^ cells) in the (C) scaffold and (D) upper leg muscle per milligram of tissue over time (*n* = 4 mice). Data are presented as means ± SD. Significance is denoted by *P ≤ 0.05 or ***P ≤ 0.001 with a two-tailed Student’s *t* test with or without Welch’s correction where applicable.

The ability of OVA-releasing PLG [poly(lactide-co-glycolide)] scaffolds to concentrate the relevant antigen-specific CD4^+^ T cells in ischemic tissue was first confirmed by implanting the scaffolds into the ischemic hindlimbs of OT-II mice (Fig. 1A). OT-II mice are genetically modified such that the T cell receptor (TCR) of their CD4^+^ T cells is antigen specific for OVA_323–339_. The OVA-containing PLG scaffolds provided sustained release of OVA over the course of at least 14 days in vitro (Fig. 1B). The numbers of antigen-specific CD4^+^ T cells in the scaffold, the adjacent ischemic upper leg muscles, and distant ischemic lower leg muscles were quantified over time (Fig. 1A and fig. S4A). The release of OVA significantly enhanced the number of CD4^+^ T cells in the scaffold at days 7, 11, and 14 after ischemic ligation compared with the blank scaffold control (Fig. 1C). In the adjacent upper leg muscles, OVA-containing scaffolds enhanced the presence of CD4^+^ T cells at days 4 and 7 after ischemic ligation compared with the blank scaffold control (Fig. 1D). Histological analysis revealed that recruited CD4^+^ T cells were concentrated in the connective tissue and skeletal muscle immediately adjacent to the OVA-containing scaffold, with fewer CD4^+^ T cells observed in the more distant skeletal muscle in the upper leg (fig. S3). OVA-containing scaffolds did not significantly increase CD4^+^ T cell recruitment in the more distant lower leg muscles, as expected (fig. S4B). In the absence of ischemia, OVA-containing scaffolds did not significantly increase the recruitment of antigen-specific CD4^+^ T cells to the OVA-containing scaffold and the adjacent upper leg muscles as compared with the blank scaffold at day 7 after scaffold implantation (fig. S4, C and D).

### OVA-releasing scaffolds induce T_H_2 response in ischemic muscle of previously immunized mice

Next, the ability of subcutaneous vaccination with OVA in the presence of aluminum hydroxide (OVA/ALUM) to generate antigen-specific T_H_2 CD4^+^ T cells in wild-type BALB/c mice was confirmed. CD4^+^ T cells isolated from the spleen of naïve or vaccinated mice were cultured in the presence of bone marrow–derived dendritic cells (BMDCs) pulsed with OVA (fig. S5A). Proliferation was found in a higher percentage of splenic CD4^+^ T cells from vaccinated mice as compared with T cells isolated from naïve mice (fig. S5, B and C), as expected. Stimulated CD4^+^ T cells from vaccinated mice secreted significantly higher levels of interleukin-5 (IL-5), a T_H_2-associated cytokine, compared with T cells from the naïve mice (fig. S5D).

**Fig. 2:**
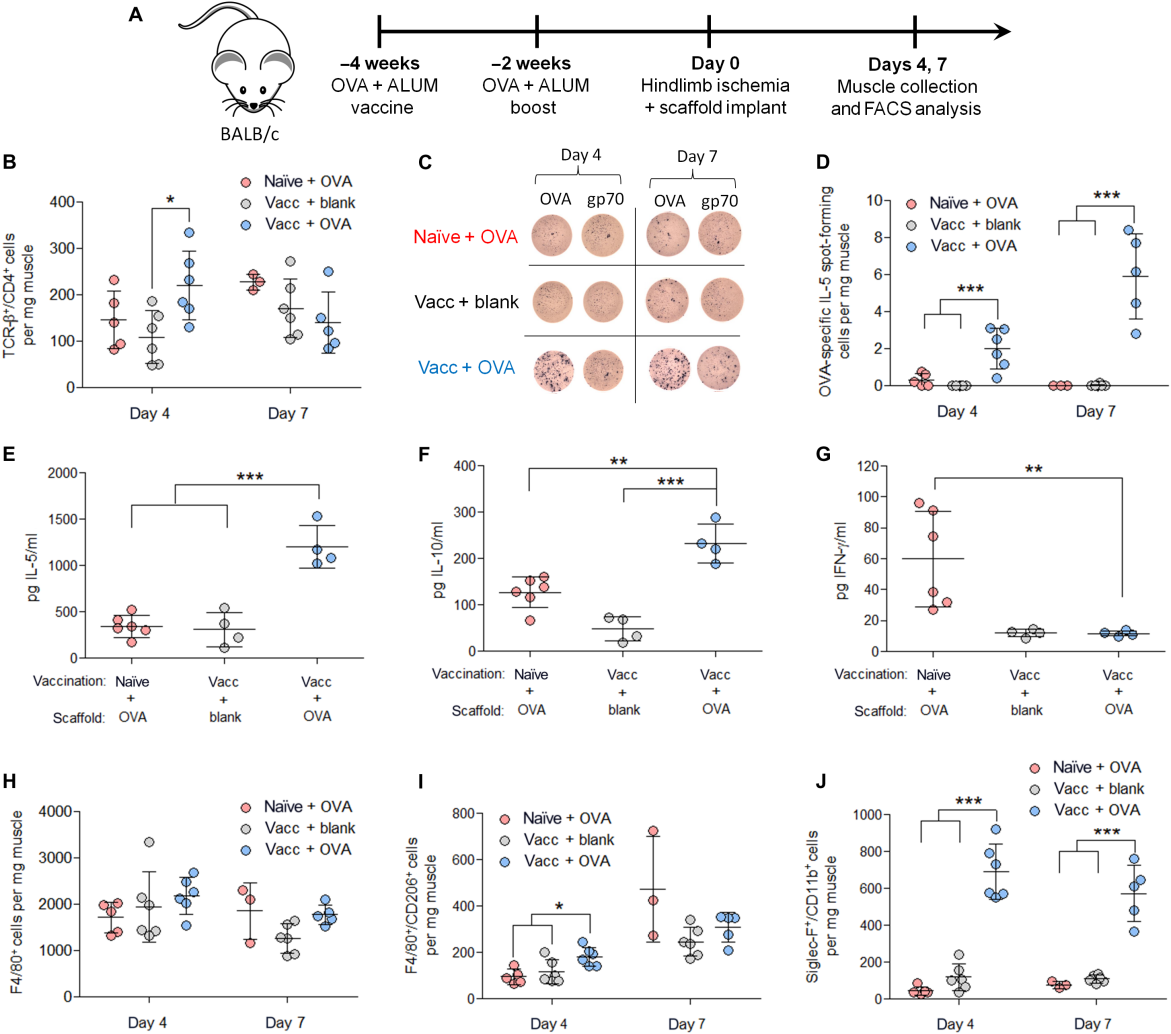
OVA-releasing scaffolds implanted in vaccinated mice enhance T_H_2 responses after ischemic injury. (**A**) Experimental setup with (left) BALB/c mice undergoing (right) timeline of procedures involving vaccination and boost with OVA/ALUM, followed by hindlimb ischemia induction and scaffold implantation. (**B** to **J**) In these figures, naïve + OVA denotes mice receiving no vaccination and implanted with an OVA-containing scaffold, vacc + blank denotes mice receiving vaccination with OVA/ALUM and a blank scaffold, and vacc + OVA denotes mice receiving vaccination with OVA/ALUM and an OVA-containing scaffold. (B) Number of CD4^+^ T cells (TCR-β^+^/CD4^+^ cells) per milligram of tissue in the ischemic upper leg at days 4 and 7 after ischemic ligation in the various treated groups. (C) Representative images of IL-5 enzyme-linked immunospot (ELISPOT) assay, where cells derived from the ischemic upper leg were cultured in the presence of splenocytes presenting either OVA or CT26-gp70 peptide as an irrelevant peptide control. (D) Quantification of OVA-specific IL-5 spot-forming cells per milligram of tissue determined by subtracting the number of IL-5 spot-forming cells in the CT26-gp70 condition from that in the OVA condition. (E to G) Concentrations of (E) IL-5, (F) IL-10, and (G) IFN-γ produced from OVA-stimulated cells isolated from the ischemic upper leg 7 days after ischemic ligation (*n* = 6 mice for naïve + OVA; *n* = 4 mice for vacc + blank; and *n* = 4 mice for vacc + OVA). (H to J) Number of (H) macrophages (F4/80^+^ cells), (I) M2a macrophages (F4/80^+^/CD206^+^ cells), and (J) eosinophils (Siglec-F^+^/CD11b^+^ cells) per milligram of tissue in the ischemic upper leg at days 4 and 7 after ischemic ligation in the various treated groups. For day 4 data in (B) to (D) and (H) to (J), *n* = 5 mice for naïve + OVA, *n* = 6 mice for vacc + blank, and *n* = 6 mice for vacc + OVA. For day 7 data in (B) to (D) and (H) to (J), *n* = 3 mice for naïve + OVA, *n* = 6 mice for vacc + blank, and *n* = 5 mice for vacc + OVA. Data are presented as means ± SD. Significance is denoted by *P ≤ 0.05, **P ≤ 0.01, or ***P ≤ 0.001 by one-way analysis of variance (ANOVA) with Bonferroni’s post hoc test.

The ability of OVA-containing scaffolds to concentrate total or antigen-specific T_H_2 CD4^+^ T cells to ischemic muscle in vaccinated mice was then tested (Fig. 2A). Three different experimental groups were tested: (i) nonvaccinated mice receiving an OVA-releasing scaffold, (ii) vaccinated mice receiving a blank scaffold, and (iii) vaccinated mice receiving an OVA-releasing scaffold. At day 4 after ischemic ligation, vaccinated mice with OVA-releasing scaffolds had significantly more total CD4^+^ T cells in the ischemic muscle than vaccinated mice with blank scaffolds (Fig. 2B); at day 7, no significant differences were observed between the tested groups (Fig. 2B). There were also no significant differences in the percent or number of total T_H_2 CD4^+^ T cells recruited to the muscle at day 4 after ischemic ligation between the tested groups (fig. S6, A and B). At day 4 and day 7, however, there was a significant increase in OVA-specific CD4^+^ T_H_2 T cells, as assayed by the number of OVA-specific IL-5 spot-forming cells, in vaccinated mice with OVA-releasing scaffolds compared with the other two tested groups (Fig. 2, C and D, and figs. S7 and S8). Ex vivo culture of cells isolated from ischemic muscle demonstrated that OVA-stimulated cells from vaccinated mice with OVA-releasing scaffolds secreted significantly higher levels of IL-5 and IL-10 compared with the two other tested groups, and lower levels of IFN-γ and granulocyte-macrophage colony-stimulating factor (GM-CSF) compared with nonvaccinated mice receiving OVA-releasing scaffolds (Fig. 2, E to G, and fig. S9A). No significant differences in IL-12p70, IL-2, tumor necrosis factor–α, or IL-4 were observed between the groups (fig. S9, B to E).

The concentrations of different innate immune cells in the ischemic thigh in response to vaccination and scaffold implantation were also quantified. No significant differences in the total number of macrophages were observed between the tested groups (Fig. 2H). The vaccinated mice with OVA-releasing scaffolds had significantly more M2a macrophages than the other two tested groups on day 4, but no differences were observed at day 7 (Fig. 2I). The vaccinated mice with OVA-releasing scaffolds also had a significantly greater number of eosinophils relative to the other two tested groups on both day 4 and day 7 (Fig. 2J). Histological analysis revealed that recruited eosinophils were concentrated in the connective tissue and skeletal muscle immediately adjacent to the scaffold, with fewer eosinophils observed in the more distant skeletal muscle in the upper leg (fig. S10).

### OVA-releasing scaffolds enhance vascular and skeletal muscle tissue recovery in ischemic muscle of vaccinated mice

**Fig. 3:**
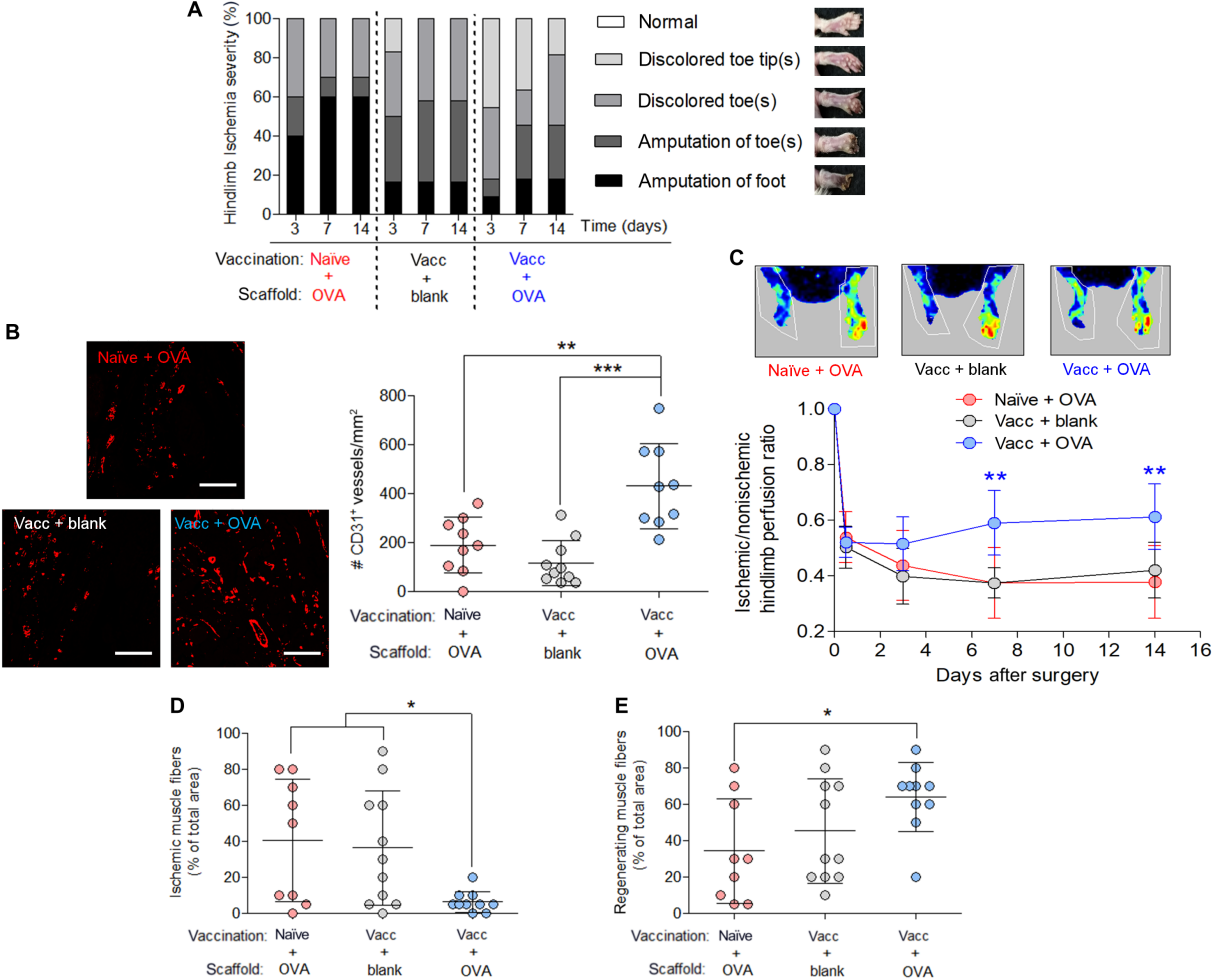
Treating vaccinated mice with OVA-releasing scaffolds enhances angiogenesis and muscle regeneration following ischemic injury. In this figure, naïve + OVA denotes mice receiving no vaccination and implanted with an OVA-containing scaffold, vacc + blank denotes mice receiving vaccination with OVA/ALUM and a blank scaffold, and vacc + OVA denotes mice receiving vaccination with OVA/ALUM and an OVA-containing scaffold. (**A**) Ischemic hindlimbs in the various treatment groups were visually examined to determine the severity of hindlimb ischemia at 3, 7, and 14 days after ischemic ligation (*n* = 10 mice for naïve + OVA, *n* = 12 mice for vacc + blank, and *n* = 11 mice for vacc + OVA). (**B**) Representative histology images and quantification of CD31^+^ blood vessel densities in ischemic tissue adjacent to scaffold and ligation site in upper leg muscles at 14 days after ischemic ligation (*n* = 9 mice for naïve + OVA, *n* = 10 mice for vacc + blank, and *n* = 9 mice for vacc + OVA). Scale bars, 100 μm. (**C**) Representative laser Doppler perfusion images and quantification of ischemic to nonischemic blood perfusion ratio in various treated mice over the course of 14 days after ischemic ligation (*n* = 9 to 10 mice for naïve + OVA, *n* = 11 to 12 mice for vacc + blank, and *n* = 10 to 11 mice for vacc + OVA). (**D** and **E**) Quantification of percent area of skeletal muscle tissue with (D) ischemic muscle fibers and (E) regenerating muscle fibers in lower leg muscles downstream of the ligation site at 14 days after ischemic ligation (*n* = 9 mice for naïve + OVA, *n* = 11 mice for vacc + blank, and *n* = 10 mice for vacc + OVA). Data are presented as means ± SD. Significance is denoted by *P ≤ 0.05, **P ≤ 0.01, and ***P ≤ 0.001 by one-way ANOVA with Bonferroni’s post hoc test (photo credit: Brian Kwee, Harvard University).

The ability of OVA-scaffold implantation to induce regeneration in vaccinated mice after ischemic injuries was next examined. By visual inspection, nonvaccinated mice with OVA-releasing scaffolds exhibited a trend of increasing severity of necrosis relative to vaccinated mice with OVA-releasing scaffolds over the course of 2 weeks after ischemic ligation (Fig. 3A). In the upper leg muscle tissue adjacent to the scaffold, the vaccinated mice with OVA-releasing scaffolds exhibited a significantly greater CD31^+^ blood vessel density compared with the other two tested groups at 2 weeks after ischemic ligation (Fig. 3B). Alpha–smooth muscle actin–positive (α-SMA^+^) blood vessels were also present in the tissue adjacent to the scaffold in all treated groups (fig. S11). The vaccinated mice with OVA-releasing scaffolds also had significantly higher blood perfusion in the ischemic limb compared with the other two tested groups (Fig. 3C). The latter result was antigen specific, as vaccinated mice implanted with a lysozyme (a control immunological peptide)–releasing scaffold exhibited greater necrosis and reduced blood perfusion relative to vaccinated mice treated with OVA-releasing scaffolds (fig. S12, A and B). The ability of OVA-scaffold implantation to enhance blood perfusion was shown to be dependent on the presence of CD4^+^ T cells. Vaccinated mice treated with OVA-releasing scaffolds and neutralizing CD4 antibodies did not show a significant increase in blood perfusion compared with vaccinated mice with blank scaffolds and nonvaccinated mice with OVA-releasing scaffolds (fig. S13). Vaccinated mice treated with OVA-releasing scaffolds and isotype control antibodies, however, did show a significant increase in blood perfusion compared with the two control groups (fig. S13). The control groups did not exhibit a marked increase in blood perfusion over time, which is characteristic of BALB/c mice undergoing hindlimb ischemia (*17*, *25*). There were also no significant differences in necrosis, CD31^+^ blood vessel density, nor blood perfusion between the nonvaccinated mice treated with OVA-releasing scaffolds and the vaccinated mice treated with blank scaffolds.

The ability of these treatments to influence myogenesis was examined by histological analysis of muscle regeneration and degeneration in the ischemic muscle. In the ischemic lower leg muscles, downstream of the ligation and scaffold implantation site, the vaccinated mice with OVA-releasing scaffolds had significantly fewer muscle fibers exhibiting coagulation (ischemic) necrosis compared with the other two tested groups (Fig. 3D and fig. S14B). Furthermore, the vaccinated mice with OVA-releasing scaffolds had significantly greater regenerating muscle fibers compared with the nonvaccinated mice with OVA-releasing scaffolds (Fig. 3E and fig. S14C).

## DISCUSSION

Implantation of antigen-releasing scaffolds effectively concentrated antigen-specific CD4^+^ T cells in the scaffold and surrounding ischemic tissue in two mouse models. These findings are consistent with previous work. The same PLG-scaffold system used in this study was previously shown to enrich antigen-specific CD4^+^ and CD8^+^ T cells inside the scaffold when providing controlled delivery of either OVA or β cell lysate subcutaneously (*24*). Similarly, persistent presentation of gp100 melanoma peptide in incomplete Freund’s adjuvant, which protects the antigen from degradation, led to sequestration of antigen-specific CD8^+^ T cells at the vaccine site (*26*). While these previous studies demonstrate concentration of antigen-specific T cells at the site of an antigen depot, the present study demonstrates concentration of antigen-specific T cells in surrounding ischemic and inflamed tissue as well.

Antigen-releasing scaffolds were also found to enhance the concentration of OVA-specific IL-5– and IL-10–secreting cells and eosinophils in the surrounding ischemic tissue of vaccinated mice; in these studies, an experimental group where mice were vaccinated with OVA alone was not tested due to the fact that subcutaneous vaccination with OVA alone in BALB/c mice can induce moderate T_H_2 responses (*27*). Vaccination in the presence of ALUM has previously been shown to induce antigen-specific IL-5 secretion from splenocytes (*23*) and to enhance TLR-induced production of IL-10 (*28*). Furthermore, chronic exposure of the lungs of OVA/ALUM-vaccinated mice to OVA, as a model of asthma, led to an increase in IL-5 levels and number of eosinophils in the resulting bronchoalveolar lavage fluid of those mice (*27*). The finding here of an enhancement in eosinophil concentration with an increased number of OVA-specific IL-5–producing cells is also consistent with the finding that IL-5 promotes eosinophil survival, is a potent eosinophil chemotactic factor, and promotes eosinophil adhesion to endothelial cells by up-regulating the leukocyte integrin CD11/18 (*29*, *30*). While M2a macrophages are associated with T_H_2 responses, we only observed a short-term increase in the presence of M2a macrophages in response to antigen-releasing scaffolds (*31*). Other T_H_2 cytokines that are implicated in M2a macrophage polarization, such as IL-4, were not up-regulated in OVA/ALUM-vaccinated CD4^+^ T cells.

Vaccinated mice treated with scaffolds releasing the antigen used for vaccination had enhanced blood perfusion in ischemic limbs, reduced foot necrosis, and an increase in angiogenesis in the fibrotic and skeletal muscle tissue adjacent to the scaffold. The increase in blood perfusion in the ischemic limb was presumably due to the formation of new vasculature adjacent to the scaffold, which bypasses the ischemic ligation, as previously described (*6*, *7*). Perfusion in the control groups decreased over time before reaching a plateau, likely due to the severity of the ligation model used in the present work. Absolute values of perfusion reported here may differ from those of other publications, as all perfusion values in these studies were recorded on mice anesthetized with ketamine and xyalzine, which induce vasoconstriction of the peripheral vasculature (*32*). Previous work using the same ischemia model and PLG system delivering angiogenic factors has demonstrated that vasculature formed at 2 weeks was stable over time due to formation of mature (α-SMA^+^) blood vessels, and significant differences in perfusion observed at 2 weeks were sustained up to 4 to 6 weeks (*6*, *7*). While perfusion was only studied up to 2 weeks in the present study, we expect stability of the vasculature and durable increases in perfusion, as α-SMA^+^ blood vessels were found here as well. The ability of this strategy to locally enhance angiogenesis is likely due, at least in part, to the increased numbers of OVA-specific IL-5– and IL-10–producing cells, which are presumably antigen-specific T_H_2 T cells and eosinophils in the ischemic thigh muscle. Previous work has shown that secreted factors from T_H_2 CD4^+^ T cells can locally enhance angiogenesis in vivo, likely by directly enhancing endothelial sprouting (*20*). The recruited eosinophils in the present study may have also contributed to angiogenesis, as they store several proangiogenic mediators, including VEGF, FGF-2, GM-CSF, IL-8, and osteopontin, in their granules (*33*, *34*), and their presence is critical to angiogenesis in mouse models of eosinophilic esophagitis (*35*). Furthermore, when OVA/ALUM-vaccinated mice were chronically challenged with OVA in the lungs, a similar increase in the number of blood vessels was observed (*36*).

Vaccinated mice treated with antigen-releasing scaffolds also demonstrated enhanced regeneration and reduced degeneration in the skeletal muscle downstream of the ligation and scaffold implantation site. This result is likely due to the restoration of blood perfusion in these skeletal muscles and not the direct enhancement of myogenesis by the scaffold implantation. Previous work has also shown that increases in blood perfusion in mice with hindlimb ischemia lead to reduced muscle atrophy (*16*), suggesting that restoration of blood perfusion alone can regulate downstream skeletal muscle degeneration. The direct impact of antigen-specific T cells on muscle regeneration requires further studies, as recent work has shown that secreted cytokines from T_H_2 T cells can directly enhance early stages of muscle regeneration (*20*) and that T_H_2 T cells are key regulators of biomaterial-based skeletal muscle regeneration (*37*). However, T_H_2 antigen-specific T cells generated from ALUM vaccination may secrete different cytokines compared with naturally derived or ex vivo–generated T_H_2 T cells.

In summary, antigen-releasing scaffolds concentrate antigen-specific CD4^+^ T cells in the scaffold and surrounding tissue. In vaccinated animals, this results in local angiogenesis, increased perfusion in ischemic limbs, and a reduction in coagulation necrosis and enhancement of regenerating myofibers in the skeletal muscle. These findings support the concept that antigen depots may provide a treatment for a variety of ischemic diseases, such as peripheral arterial disease and diabetic ulcers, in patients previously vaccinated with aluminum adjuvants. Clinically, this therapy may be capable of exploiting aluminum-containing childhood vaccines, such as DTaP, which the Centers for Disease Control and Prevention recommends all persons in the United States receive multiple times during childhood and every 10 years during adulthood. Future work will be required to explore the impact of different numbers of vaccination boosts and the effects at longer time points after vaccination to model humans that have been vaccinated years to decades earlier. Furthermore, scaling up this treatment to the length scale of ischemic human limbs will likely require multiple scaffold implantations to account for diffusion limitations of the antigen. More broadly, these findings demonstrate the novel idea that memory T cells can be therapeutically used to promote vascularization and support the premise that biomaterials can manipulate T cells to enhance regeneration.

## MATERIALS AND METHODS

### Immunization

All animal work was performed in compliance with the National Institutes of Health (NIH) and institutional guidelines. Female BALB/c mice (the Jackson Laboratory, catalog no. 000651) at 5 to 7 weeks old were immunized by the axillary lymph nodes with a subcutaneous injection of 250 μg of OVA protein (InvivoGen, catalog no. vac-pova) mixed in a 1:1 ratio with alhydrogel adjuvant 2% (aluminum hydroxide gel, ALUM; InvivoGen, catalog no. vac-alu-250). Mice were boosted 2 weeks later with the same formulation of OVA and ALUM with a subcutaneous injection by the inguinal lymph nodes. These vaccinations were done on the side of the mouse that is opposite to the site of ischemic ligation.

### Restimulation of splenic CD4^+^ T cells with OVA

To isolate BMDCs, bone marrow cells were first isolated from the femur and tibia bones of unimmunized BALB/c mice. These cells were then cultured in RPMI 1640 media (Sigma-Aldrich, catalog no. 6504) supplemented with 10% heat-inactivated fetal bovine serum (FBS) (Sigma-Aldrich, catalog no. F4135), 1% penicillin-streptomycin (Gibco, catalog no. 15140-122), 50 μM β-mercaptoethanol (Sigma-Aldrich, catalog no. M7522), and murine GM-CSF (20 ng/ml; PeproTech, catalog no. 315-03). On day 6 of culture, cells were pulsed with lipopolysaccharide (100 ng/ml; Sigma-Aldrich, catalog no. L5293) for 24 hours. On day 7, BMDCs were then collected and pulsed with OVA (20 μg/ml). Separately, CD4^+^ T cells were isolated from spleens of vaccinated mice 8 days after the boost by first physically grinding the spleens onto a 70-μm cell filter in phosphate-buffered saline (PBS) to obtain splenocytes. Red blood cells (RBCs) were lysed using RBC lysis buffer (BioLegend, catalog no. 420301), and CD4^+^ T cells were obtained using the MACS CD4^+^ T Cell Isolation Kit (Miltenyi Biotec, catalog no. 130-104-454). The sorted CD4 T cells were labeled with carboxyfluorescein diacetate succinimidyl ester (CFSE) (BioLegend, catalog no. 423801) to track cell proliferation and were cultured with the OVA-pulsed BMDCs at an 8:1 (T-cell:dendritic cell) ratio for 3 days at 37°C to allow for antigen-specific T cell proliferation. On day 3, cells were collected and prepared for fluorescence-activated cell sorting (FACS) analysis. Cells were treated with Fc block (BioLegend, anti-mouse CD16/32, catalog no. 101320) and stained with the following antibodies (manufacturer, clone name, and flurophore in parenthesis): anti-CD4 [BioLegend, GK1.5, phycoerythrin (PE)/Cy7] and anti-CD3 (BioLegend, 17A2, APC/Fire 750). Live cells were determined with fixable viability dye eFluor450 (eBioscience, catalog no. 65-0863-14). Media supernatants were also collected on day 3 to analyze IL-5 secretion by an enzyme-linked immunosorbent assay (R&D Systems, catalog no. DY405).

### Scaffold fabrication

OVA-loaded PLG scaffolds were fabricated as previously described (*24*). Briefly, 30-μm LG30K PLG microspheres (Phosphorex, catalog no. LG30-8515; 18 mg per scaffold) were mixed with OVA protein in Milli-Q water at 1 mg of OVA and 1 ml of Milli-Q water per scaffold. After vortexing the samples and allowing the OVA to adsorb to the microspheres for 15 min at room temperature, samples were vortexed again and snap frozen in liquid nitrogen. Samples were then lyophilized for at least 4 days. The resulting OVA/microsphere powder was mixed with 130 mg of sucrose (sieved to 250- to 425-μm particle size) per scaffold and compression molded into 8-mm-diameter discs with a Carver Model 3850 manual press at 1500 psi for 45 s. The compressed discs were placed in a pressure cylinder and exposed to 800 psi CO_2_. Scaffolds were left overnight, and then the pressure was slowly released over 1 to 2 min to allow the polymer particles to fuse and expand. Scaffolds were kept at −20°C until use. Before implantation, scaffolds were placed in 5 ml of distilled water to leach sucrose, yielding scaffolds that were 85 to 94% porous. For lysozyme-loaded scaffolds, the same process was followed using lysozyme (Sigma-Aldrich, catalog no. L6876) run through Pierce High Capacity Endotoxin Removal Spin Columns (Thermo Fisher Scientific, catalog no. 88274) instead of OVA.

### OVA release from scaffolds

The release of OVA from scaffolds was measured by placing the scaffolds in 3 ml of PBS in an incubator at 37°C. At various time points, the PBS was collected and replaced. The amount of OVA in the PBS was measured with the Pierce Micro BCA Protein Assay Kit (Thermo Fisher Scientific, catalog no. 23235). For each individual scaffold, a release curve of total OVA released versus time was plotted on GraphPad Prism 5.0 (GraphPad Software Inc., CA), and a curve-fitted two-phase exponential association equation was calculated using nonlinear regression. The instantaneous release of OVA at specific days for each scaffold was determined by taking the derivative of the equation.

### Hindlimb ischemia

Female B6.Cg-Tg(TcraTcrb)425bn/J mice (OT-II mice, the Jackson Laboratory, catalog no. 004194) or BALB/c mice 6 to 12 weeks were used for these studies. Mice were anesthetized with an intraperitoneal injection of a mixture of ketamine (120 mg kg^−1^) and xylazine (10 mg kg^−1^) before all surgical procedures. Hindlimb ischemia was induced by unilateral external iliac and femoral artery and vein ligation with 5-0 Ethilon sutures, as previously described (*38*). Immediately after vessel ligation, a PLG scaffold (8 mm in diameter and approximately 2 mm thick) was placed on top of the ligation site, covering both the external iliac and femoral artery and vein, as previously described, to induce new blood vessel formation to bypass the ischemic ligation and restore blood perfusion to ischemic limbs (fig. S1) (*6*, *7*, *39*, *40*). In the no ischemia control condition experiments, PLG scaffolds were placed subcutaneously on top of the external iliac and femoral artery and vein without vessel ligation. PLG scaffolds were not placed by the distal gastrocnemius muscle in any studies due to the concern that areas of pronounced ischemia lack sufficient functional blood vessels and collaterals to recruit circulating antigen-specific T cells.

All measurements of the blood perfusion in the ischemic limbs were performed on anesthetized mice using a laser Doppler perfusion imaging (LDPI) analyzer (PeriScan PIM II, Perimed AB, Sweden), as previously described (*7*, *41*). Mice were anesthetized with a mixture of ketamine (120 mg kg^−1^) and xylazine (10 mg kg^−1^) and laid out in the supine position on day 0 and in the prone position on remaining days on a heating pad at 37°C, with palms of the feet facing up. Perfusion data were obtained by scanning both hindlimbs from the toe tips up to the lower part of the gastrocnemius muscle in the dark. Quantitative analysis of LDPI images was performed on LDPIwin 2.5 software, and the perfusion value in the ischemic limbs was calculated as the ratio of the ischemic to nonischemic limb blood perfusion, as previously described (*7*, *42*–*45*). If necrosis and autoamputation in the ischemic limb extended into the regio tarsi of the hindlimb during the study, then mice were euthanized and removed from subsequent analysis in accordance with the Harvard University Faculty of Arts and Sciences Institutional Animal Care and Use Committee guidelines.

### Extraction and flow cytometry of leukocytes from scaffolds and muscle

Scaffolds and muscle from the hindlimbs were excised from euthanized mice for isolation of infiltrating leukocytes. All muscle groups in the thigh of the hindlimb were collected and labeled as “upper leg muscles.” All muscle groups below the knee and above the foot of the hindlimb were collected and labeled as “lower leg muscles.” Single-cell suspensions from scaffolds and ischemic muscle were prepared by first mincing tissues, followed by digestion in Dulbecco’s modified Eagle’s medium (DMEM) media with collagenase II (250 U/ml; Gibco, catalog no. 17101015) and deoxyribonuclease I (DNaseI) (150 μg/ml; Sigma-Aldrich, catalog no. DN25) for 30 min at 37°C. Resulting cell and tissue suspensions were poured over and mashed on a 70-μm cell filter and were washed with DMEM media supplemented with 10% heat-inactivated FBS. Debris from scaffolds and tissue were removed using Lympholyte-M (Cedarlane, catalog no. CL5035), and RBCs were lysed with RBC lysis buffer (BioLegend, catalog no. 420301) according to the manufacturer’s guidelines. Isolated cells were treated with Fc block (BioLegend, anti-mouse CD16/32, catalog no. 101320) and stained with the following antibodies (manufacturer, clone name, and flurophor in parenthesis): CD45 (BioLegend, 30-F11, PE), TCR-β chain [BioLegend, H57-597 fluorescein isothiocyanate (FITC)], CD4 (BioLegend, GK1.5, PE/Cy7), GATA3 [BioLegend, 16E10A23, allophycocyanin (APC)], F4/80 (BioLegend, BM8, APC), CD206 (BioLegend, C068C2, FITC), CD11b (BioLegend, Pacific Blue, M1/70), and Siglec-F (BD Pharmingen, E50-2440, PerCP-Cy5.5). Live cells were determined with fixable viability dye eFluor780 (eBioscience, catalog no. 65-0865-14). To determine the numbers of different immune cell types, the number of live cells from the samples was counted using a Countess Automated Cell Counter (Thermo Fisher Scientific), and this was multiplied by the percentage of live cells of each cell type from the FACS analysis.

### Enzyme-linked immunospot analysis

Mouse IL-5 enzyme-linked immunospot (ELISPOT) assays (R&D Systems, catalog no. EL405) were performed according to the manufacturer’s guidelines. Assays were performed in serum-free AIM V medium (Life Technologies, catalog no. 12055-091). Cells (2.5 × 10^5^) isolated from the ischemic thigh of mice were cultured with splenocytes (2.5 × 10^5^) isolated from naïve mice in the presence of OVA protein (10 μg/ml; InvivoGen, catalog no. vac-pova) or ct26-gp70 peptide (10 μg/ml; Peptide 2.0, peptide sequence SPSYAYHQF) as an irrelevant peptide control. ELISPOT assays were counted and analyzed by Immunospot ScAnalysis Services. To quantify the number of cells that are OVA-specific IL-5 spot-forming cells, the number of IL-5 spot-forming cells in the ct26-gp70 peptide–stimulated condition was subtracted from the number of IL-5 spot-forming cells in the OVA-stimulated condition.

### Cytokine immunoassay

Bio-Plex Pro Mouse Cytokine T_H_1/T_H_2 Assay (Bio-Rad, catalog no. m6000003j7) was performed according to the manufacturer’s guidelines. Cells (2.5 × 10^5^) isolated from the ischemic thighs of mice were cultured with splenocytes (2.5 × 10^5^) isolated from naïve mice in serum-free AIM V medium in the presence of OVA protein (20 μg/ml) or ct26-gp70 peptide (20 μg/ml) as an irrelevant peptide control. After 3 days of culture, media supernatants were collected and analyzed on a BioPlex 3D System (Bio-Rad). To determine OVA-specific cytokine production, the concentration of cytokines detected in the ct26-gp70 peptide condition was subtracted from the OVA protein condition.

### Histological analysis

Ischemic calf muscles and thigh muscles with implanted PLG scaffold were retrieved at 7 or 14 days after ischemia induction, fixed, and prepared for sectioning. Muscles were either frozen in optimal cutting temperature compound (OCT) or paraffin embedded and sectioned by the Rodent Histopathology Core at Dana-Farber/Harvard Cancer Center in Boston, MA. Ischemic calf muscle sections were stained with hematoxylin and eosin and were graded on percentage of total muscle area that exhibited coagulation (ischemic) necrosis or regenerating myofibers by a pathologist in a blinded fashion. The remaining regions of muscle area consisted of normal muscle fibers, inflammation, and fatty replacement of muscle fibers. Ischemic muscle fibers were distinguished as fragmented muscle fibers that were paler in color compared with normal muscle fibers. Regenerating muscle fibers were distinguished by centrally nucleated muscle fibers. Ischemic thigh muscle and PLG scaffold were stained for CD31, α-SMA, CD4, or Siglec-F prior to mounting the slide with ProLong Gold Antifade Mountant with DAPI (4′,6-diamidino-2-phenylindole) (Thermo Fisher Scientific, catalog no. P36941). For CD31 staining, sections underwent antigen retrieval with proteinase K (Sigma-Aldrich, catalog no. P2308) and were stained with the TSA Plus TMR System (PerkinElmer, catalog no. NEL 742001), according to the manufacturer’s protocol, using a rat anti-CD31 primary antibody (BD Pharminogen, catalog no. 557355) and a horseradish peroxidase anti-rat secondary antibody (InvitroGen, catalog no. 31470). For α-SMA staining, sections underwent heat-mediated antigen retrieval in Dako Cytomation Target Retrieval Solution (pH 9) and were stained with the mouse anti–α-SMA primary antibody (Abcam, catalog no. ab7817) and the anti-mouse Alexa Fluor 647 secondary antibody (LifeTech, catalog no. A21235). For CD4 staining, sections underwent heat-mediated antigen retrieval in citrate buffer and were stained with the rabbit anti-CD4 primary antibody (Abcam, catalog no. ab183685) and the anti-rabbit Alexa Fluor 555 secondary antibody (LifeTech, catalog no. A-21428). For Siglec-F staining, sections underwent antigen retrieval with proteinase K (Sigma-Aldrich, catalog no. P2308) and were stained with the rat anti–Siglec-F primary antibody (eBioscience, catalog no. 14-1702-82) and the anti-rat Alexa Fluor 594 secondary antibody (LifeTech, catalog no. A-21209).

Sections from each sample were visualized with a Zeiss LSM confocal microscope. Images of the ischemic skeletal muscle immediately adjacent to the PLG scaffold were taken, and the number of CD31 blood vessels in each image was determined using the ImageJ Particle Analysis tool.

### CD4^+^ T cell depletion

CD4^+^ T cells were depleted by intraperitoneal injection of 400 μg of anti-CD4 monoclonal antibody (BioXCell, catalog no. BE0003-1, clone GK1.5) twice weekly, starting 3 days before vaccination. Control mice received an intraperitoneal injection of rat IgG2b isotype control antibody (BioXCell, catalog no. BE0090, clone LTF-2). CD4^+^ T cell depletion was confirmed using flow cytometry.

### Statistics

All values in the present study are expressed as means ± SD. Data were statistically analyzed in GraphPad Prism 5.0 (GraphPad Software Inc., CA). Significance between groups was determined using analyses of variance (ANOVAs) with Bonferroni’s post hoc test in experiments containing more than two groups. In experiments containing two groups, significance was determined using unpaired, two-tailed *t* tests, with Welch’s correction when SDs were significantly different. Sample variance was tested using the *F* test. Statistical significance was determined at *P* ≤ 0.05.

## Supplementary Material

http://advances.sciencemag.org/cgi/content/full/5/7/eaav6313/DC1

Download PDF

## SUPPLEMENTARY MATERIALS

Supplementary material for this article is available at http://advances.sciencemag.org/cgi/content/full/5/7/eaav6313/DC1 Fig. S1. Scaffold implantation on ischemic ligation. Fig. S2. Representative FACS gating strategy for quantifying percentage and number of different immune cells. Fig. S3. Distribution of CD4^+^ T cells recruited to scaffold and upper leg muscles. Fig. S4. Recruitment of CD4^+^ T cells in OT-II mice. Fig. S5. OVA/ALUM vaccination enhances IL-5–producing OVA-specific CD4^+^ T cells in BALB/c mice. Fig. S6. Concentration of T_H_2 CD4^+^ T cells in ischemic hindlimb muscle. Fig. S7. Images of wells from IL-5 ELISPOT assay, measuring IL-5–secreting cells from cells isolated from ischemic thighs 4 days after ischemic ligation. Fig. S8. Images of wells from IL-5 ELISPOT assay, measuring IL-5–secreting cells from cells isolated from ischemic thighs 7 days after ischemic ligation. Fig S9. Concentration of T_H_1/T_H_2 cytokines secreted by OVA-stimulated cells in ischemic hindlimb muscle. Fig. S10. Distribution of eosinophils recruited to scaffold and upper leg muscles. Fig. S11. Presence of α-SMA^+^ blood vessels in tissue adjacent to scaffold. Fig. S12. Antigen-releasing scaffolds enhance blood perfusion recovery following ischemic injury in an antigen-specific manner. Fig. S13. Blood perfusion recovery in vaccinated mice with OVA-releasing scaffolds depends on the presence of CD4^+^ T cells. Fig. S14. Characterization of types of muscle fibers in histological sections of ischemic lower leg muscles.

## References

[1] Ouriel K., Peripheral arterial disease. Lancet 358, 1257–1264 (2001). 11675083 10.1016/S0140-6736(01)06351-6

[2] Isner J. M., Asahara T., Angiogenesis and vasculogenesis as therapeutic strategies for postnatal neovascularization. J. Clin. Invest. 103, 1231–1236 (1999). 10225965 10.1172/JCI6889 PMC408362

[3] Annex B. H., Therapeutic angiogenesis for critical limb ischaemia. Nat. Rev. Cardiol. 10, 387–396 (2013). 23670612 10.1038/nrcardio.2013.70

[4] Simons M., Bonow R. O., Chronos N. A., Cohen D. J., Giordano F. J., Hammond H. K., Laham R. J., Li W., Pike M., Sellke F. W., Stegmann T. J., Udelson J. E., Rosengart T. K., Clinical trials in coronary angiogenesis: Issues, problems, consensus: An expert panel summary. Circulation 102, e73–e86 (2000). 10982554 10.1161/01.cir.102.11.e73

[5] Cao L., Mooney D. J., Spatiotemporal control over growth factor signaling for therapeutic neovascularization. Adv. Drug Deliv. Rev. 59, 1340–1350 (2007). 17868951 10.1016/j.addr.2007.08.012 PMC2581871

[6] Sun Q., Chen R. R., Shen Y., Mooney D. J., Rajagopalan S., Grossman P. M., Sustained vascular endothelial growth factor delivery enhances angiogenesis and perfusion in ischemic hind limb. Pharm. Res. 22, 1110–1116 (2005). 16028011 10.1007/s11095-005-5644-2

[7] Chen R. R., Snow J. K., Palmer J. P., Lin A. S., Duvall C. L., Guldberg R. E., Mooney D. J., Host immune competence and local ischemia affects the functionality of engineered vasculature. Microcirculation 14, 77–88 (2007). 17365663 10.1080/10739680601131101

[8] Heil M., Ziegelhoeffer T., Pipp F., Kostin S., Martin S., Clauss M., Schaper W., Blood monocyte concentration is critical for enhancement of collateral artery growth. Am. J. Physiol. Heart Circ. Physiol. 283, H2411–H2419 (2002). 12388258 10.1152/ajpheart.01098.2001

[9] Roh J. D., Sawh-Martinez R., Brennan M. P., Jay S. M., Devine L., Rao D. A., Yi T., Mirensky T. L., Nalbandian A., Udelsman B., Hibino N., Shinoka T., Saltzman W. M., Snyder E., Kyriakides T. R., Pober J. S., Breuer C. K., Tissue-engineered vascular grafts transform into mature blood vessels via an inflammation-mediated process of vascular remodeling. Proc. Natl. Acad. Sci. U.S.A. 107, 4669–4674 (2010). 20207947 10.1073/pnas.0911465107 PMC2842056

[10] Spiller K. L., Anfang R. R., Spiller K. J., Ng J., Nakazawa K. R., Daulton J. W., Vunjak-Novakovic G., The role of macrophage phenotype in vascularization of tissue engineering scaffolds. Biomaterials 35, 4477–4488 (2014). 24589361 10.1016/j.biomaterials.2014.02.012 PMC4000280

[11] Takeda Y., Costa S., Delamarre E., Roncal C., Leite de Oliveira R., Squadrito M. L., Finisguerra V., Deschoemaeker S., Bruyère F., Wenes M., Hamm A., Serneels J., Magat J., Bhattacharyya T., Anisimov A., Jordan B. F., Alitalo K., Maxwell P., Gallez B., Zhuang Z. W., Saito Y., Simons M., de Palma M., Mazzone M., Macrophage skewing by Phd2 haplodeficiency prevents ischaemia by inducing arteriogenesis. Nature 479, 122–126 (2011). 21983962 10.1038/nature10507 PMC4659699

[12] Kwee B. J., Mooney D. J., Manipulating the intersection of angiogenesis and inflammation. Ann. Biomed. Eng. 43, 628–640 (2015). 25316589 10.1007/s10439-014-1145-y PMC4380659

[13] Spiller K. L., Freytes D. O., Vunjak-Novakovic G., Macrophages modulate engineered human tissues for enhanced vascularization and healing. Ann. Biomed. Eng. 43, 616–627 (2015). 25331098 10.1007/s10439-014-1156-8 PMC4380684

[14] Awojoodu A. O., Ogle M. E., Sefcik L. S., Bowers D. T., Martin K., Brayman K. L., Lynch K. R., Peirce-Cottler S. M., Botchwey E., Sphingosine 1-phosphate receptor 3 regulates recruitment of anti-inflammatory monocytes to microvessels during implant arteriogenesis. Proc. Natl. Acad. Sci. U.S.A. 110, 13785–13790 (2013). 23918395 10.1073/pnas.1221309110 PMC3752259

[15] Spiller K. L., Nassiri S., Witherel C. E., Anfang R. R., Ng J., Nakazawa K. R., Yu T., Vunjak-Novakovic G., Sequential delivery of immunomodulatory cytokines to facilitate the M1-to-M2 transition of macrophages and enhance vascularization of bone scaffolds. Biomaterials 37, 194–207 (2015). 25453950 10.1016/j.biomaterials.2014.10.017 PMC4312192

[16] Stabile E., Burnett M. S., Watkins C., Kinnaird T., Bachis A., la Sala A., Miller J. M., Shou M., Epstein S. E., Fuchs S., Impaired arteriogenic response to acute hindlimb ischemia in CD4-knockout mice. Circulation 108, 205–210 (2003). 12821542 10.1161/01.CIR.0000079225.50817.71

[17] van Weel V., Toes R. E., Seghers L., Deckers M. M., de Vries M. R., Eilers P. H., Sipkens J., Schepers A., Eefting D., van Hinsbergh V. W., van Bockel J. H., Quax P. H., Natural killer cells and CD4+ T-cells modulate collateral artery development. Arterioscler. Thromb. Vasc. Biol. 27, 2310–2318 (2007). 17717295 10.1161/ATVBAHA.107.151407

[18] Couffinhal T., Silver M., Kearney M., Sullivan A., Witzenbichler B., Magner M., Annex B., Peters K., Isner J. M., Impaired collateral vessel development associated with reduced expression of vascular endothelial growth factor in ApoE^−/−^ mice. Circulation 99, 3188–3198 (1999). 10377084 10.1161/01.cir.99.24.3188

[19] Blotnick S., Peoples G. E., Freeman M. R., Eberlein T. J., Klagsbrun M., T lymphocytes synthesize and export heparin-binding epidermal growth factor-like growth factor and basic fibroblast growth factor, mitogens for vascular cells and fibroblasts: Differential production and release by CD4+ and CD8+ T cells. Proc. Natl. Acad. Sci. U.S.A. 91, 2890–2894 (1994). 7909156 10.1073/pnas.91.8.2890 PMC43479

[20] Kwee B. J., Budina E., Najibi A. J., Mooney D. J., CD4 T-cells regulate angiogenesis and myogenesis. Biomaterials 178, 109–121 (2018). 29920403 10.1016/j.biomaterials.2018.06.003 PMC6090550

[21] Puxeddu I., Alian A., Piliponsky A. M., Ribatti D., Panet A., Levi-Schaffer F., Human peripheral blood eosinophils induce angiogenesis. Int. J. Biochem. Cell Biol. 37, 628–636 (2005). 15618019 10.1016/j.biocel.2004.09.001

[22] Mitkus R. J., King D. B., Hess M. A., Forshee R. A., Walderhaug M. O., Updated aluminum pharmacokinetics following infant exposures through diet and vaccination. Vaccine 29, 9538–9543 (2011). 22001122 10.1016/j.vaccine.2011.09.124

[23] Brewer J. M., Conacher M., Hunter C. A., Mohrs M., Brombacher F., Alexander J., Aluminium hydroxide adjuvant initiates strong antigen-specific Th2 responses in the absence of IL-4- or IL-13-mediated signaling. J. Immunol. 163, 6448–6454 (1999). 10586035

[24] Thelin M. A., Kissler S., Vigneault F., Watters A. L., White D., Koshy S. T., Vermillion S. A., Mooney D. J., Serwold T., Ali O. A., In vivo enrichment of diabetogenic T cells. Diabetes 66, 2220–2229 (2017). 28396510 10.2337/db16-0946 PMC5521861

[25] Scholz D., Ziegelhoeffer T., Helisch A., Wagner S., Friedrich C., Podzuweit T., Schaper W., Contribution of arteriogenesis and angiogenesis to postocclusive hindlimb perfusion in mice. J. Mol. Cell. Cardiol. 34, 775–787 (2002). 12099717 10.1006/jmcc.2002.2013

[26] Hailemichael Y., Dai Z., Jaffarzad N., Ye Y., Medina M. A., Huang X. F., Dorta-Estremera S. M., Greeley N. R., Nitti G., Peng W., Liu C., Lou Y., Wang Z., Ma W., Rabinovich B., Sowell R. T., Schluns K. S., Davis R. E., Hwu P., Overwijk W. W., Persistent antigen at vaccination sites induces tumor-specific CD8^+^ T cell sequestration, dysfunction and deletion. Nat. Med. 19, 465–472 (2013). 23455713 10.1038/nm.3105 PMC3618499

[27] Conrad M. L., Yildirim A. Ö., Sonar S. S., Kılıç A., Sudowe S., Lunow M., Teich R., Renz H., Garn H., Comparison of adjuvant and adjuvant-free murine experimental asthma models. Clin. Exp. Allergy 39, 1246–1254 (2009). 19438585 10.1111/j.1365-2222.2009.03260.x PMC2728898

[28] Li H., Nookala S., Re F., Aluminum hydroxide adjuvants activate caspase-1 and induce IL-1β and IL-18 release. J. Immunol. 178, 5271–5276 (2007). 17404311 10.4049/jimmunol.178.8.5271

[29] Yamaguchi Y., Hayashi Y., Sugama Y., Miura Y., Kasahara T., Kitamura S., Torisu M., Mita S., Tominaga A., Takatsu K., Highly purified murine interleukin 5 (IL-5) stimulates eosinophil function and prolongs in vitro survival. IL-5 as an eosinophil chemotactic factor. J. Exp. Med. 167, 1737–1742 (1988). 2835420 10.1084/jem.167.5.1737 PMC2188945

[30] Walsh G., Hartnell A., Wardlaw A. J., Kurihara K., Sanderson C. J., Kay A. B., IL-5 enhances the in vitro adhesion of human eosinophils, but not neutrophils, in a leucocyte integrin (CD11/18)-dependent manner. Immunology 71, 258–265 (1990). 2228026 PMC1384313

[31] Mantovani A., Sica A., Sozzani S., Allavena P., Vecchi A., Locati M., The chemokine system in diverse forms of macrophage activation and polarization. Trends Immunol. 25, 677–686 (2004). 15530839 10.1016/j.it.2004.09.015

[32] Greco A., Ragucci M., Liuzzi R., Gargiulo S., Gramanzini M., Coda A. R., Albanese S., Mancini M., Salvatore M., Brunetti A., Repeatability, reproducibility and standardisation of a laser Doppler imaging technique for the evaluation of normal mouse hindlimb perfusion. Sensors 13, 500–515 (2013). 10.3390/s130100500 PMC3574687 23275085

[33] Puxeddu I., Ribatti D., Crivellato E., Levi-Schaffer F., Mast cells and eosinophils: A novel link between inflammation and angiogenesis in allergic diseases. J. Allergy Clin. Immunol. 116, 531–536 (2005). 16159620 10.1016/j.jaci.2005.06.007

[34] Puxeddu I., Berkman N., Ribatti D., Bader R., Haitchi H. M., Davies D. E., Howarth P. H., Levi-Schaffer F., Osteopontin is expressed and functional in human eosinophils. Allergy 65, 168–174 (2010). 19804447 10.1111/j.1398-9995.2009.02148.x

[35] Rubinstein E., Cho J. Y., Rosenthal P., Chao J., Miller M., Pham A., Aceves S. S., Varki A., Broide D. H., Siglec-F inhibition reduces esophageal eosinophilia and angiogenesis in a mouse model of eosinophilic esophagitis. J. Pediatr. Gastroenterol. Nutr. 53, 409–416 (2011). 21970996 10.1097/MPG.0b013e3182182ff8 PMC3980963

[36] Asosingh K., Swaidani S., Aronica M., Erzurum S. C., Th1-and Th2-dependent endothelial progenitor cell recruitment and angiogenic switch in asthma. J. Immunol. 178, 6482–6494 (2007). 17475878 10.4049/jimmunol.178.10.6482

[37] Sadtler K., Estrellas K., Allen B. W., Wolf M. T., Fan H., Tam A. J., Patel C. H., Luber B. S., Wang H., Wagner K. R., Powell J. D., Housseau F., Pardoll D. M., Elisseeff J. H., Developing a pro-regenerative biomaterial scaffold microenvironment requires T helper 2 cells. Science 352, 366–370 (2016). 27081073 10.1126/science.aad9272 PMC4866509

[38] Silva E. A., Mooney D., Spatiotemporal control of vascular endothelial growth factor delivery from injectable hydrogels enhances angiogenesis. J. Thromb. Haemost. 5, 590–598 (2007). 17229044 10.1111/j.1538-7836.2007.02386.x

[39] Yuen W. W., Du N. R., Chan C. H., Silva E. A., Mooney D. J., Mimicking nature by codelivery of stimulant and inhibitor to create temporally stable and spatially restricted angiogenic zones. Proc. Natl. Acad. Sci. U.S.A. 107, 17933–17938 (2010). 20921366 10.1073/pnas.1001192107 PMC2964247

[40] Silva E. A., Kim E.-S., Kong H. J., Mooney D. J., Material-based deployment enhances efficacy of endothelial progenitor cells. Proc. Natl. Acad. Sci. 105, 14347–14352 (2008). 18794520 10.1073/pnas.0803873105 PMC2567164

[41] Raimondo T. M., Mooney D. J., Functional muscle recovery with nanoparticle-directed M2 macrophage polarization in mice. Proc. Natl. Acad. Sci. U.S.A. 115, 10648–10653 (2018). 30275293 10.1073/pnas.1806908115 PMC6196479

[42] Borselli C., Cezar C. A., Shvartsman D., Vandenburgh H. H., Mooney D. J., The role of multifunctional delivery scaffold in the ability of cultured myoblasts to promote muscle regeneration. Biomaterials 32, 8905–8914 (2011). 21911253 10.1016/j.biomaterials.2011.08.019 PMC3210474

[43] Cezar C. A., Roche E. T., Vandenburgh H. H., Duda G. N., Walsh C. J., Mooney D. J., Biologic-free mechanically induced muscle regeneration. Proc. Natl. Acad. Sci. U.S.A. 113, 1534–1539 (2016). 26811474 10.1073/pnas.1517517113 PMC4760832

[44] Tan Y., Shao H., Eton D., Yang Z., Alonso-Diaz L., Zhang H., Schulick A., Livingstone A. S., Yu H., Stromal cell-derived factor-1 enhances pro-angiogenic effect of granulocyte-colony stimulating factor. Cardiovasc. Res. 73, 823–832 (2007). 17258698 10.1016/j.cardiores.2006.12.015 PMC2243257

[45] Yin K.-J., Olsen K., Hamblin M., Zhang J., Schwendeman S. P., Chen Y. E., Vascular endothelial cell-specific microRNA-15a inhibits angiogenesis in hindlimb ischemia. J. Biol. Chem. 287, 27055–27064 (2012). 22692216 10.1074/jbc.M112.364414 PMC3411046

