# Supplementary material for: Treating ischemia via recruitment of antigen-specific T cells
Source: Sci Adv. 2019 Jul 31;5(7):eaav6313. doi: 10.1126/sciadv.aav6313 (PMC6669016; doi:10.1126/sciadv.aav6313)
Supplement: http://advances.sciencemag.org/cgi/content/full/5/7/eaav6313/DC1 [file supp_5_7_eaav6313__index.html]

Science Advances | Science AdvancesAAASSearchScience AdvancesMenu

## Supplementary Materials

**This PDF file includes:**

- Fig. S1. Scaffold implantation on ischemic ligation.
- Fig. S2. Representative FACS gating strategy for quantifying percentage and number of different immune cells.
- Fig. S3. Distribution of CD4+ T cells recruited to scaffold and upper leg muscles.
- Fig. S4. Recruitment of CD4+ T cells in OT-II mice.
- Fig. S5. OVA/ALUM vaccination enhances IL-5–producing OVA-specific CD4+ T cells in BALB/c mice.
- Fig. S6. Concentration of TH2 CD4+ T cells in ischemic hindlimb muscle.
- Fig. S7. Images of wells from IL-5 ELISPOT assay, measuring IL-5–secreting cells from cells isolated from ischemic thighs 4 days after ischemic ligation.
- Fig. S8. Images of wells from IL-5 ELISPOT assay, measuring IL-5–secreting cells from cells isolated from ischemic thighs 7 days after ischemic ligation.
- Fig S9. Concentration of TH1/TH2 cytokines secreted by OVA-stimulated cells in ischemic hindlimb muscle.
- Fig. S10. Distribution of eosinophils recruited to scaffold and upper leg muscles.
- Fig. S11. Presence of α-SMA+ blood vessels in tissue adjacent to scaffold.
- Fig. S12. Antigen-releasing scaffolds enhance blood perfusion recovery following ischemic injury in an antigen-specific manner.
- Fig. S13. Blood perfusion recovery in vaccinated mice with OVA-releasing scaffolds depends on the presence of CD4+ T cells.
- Fig. S14. Characterization of types of muscle fibers in histological sections of ischemic lower leg muscles.

Download PDF

**Files in this Data Supplement:**

- Adobe PDF - aav6313\_SM.pdf
