# Supplementary material for: Treating ischemia via recruitment of antigen-specific T cells
Source: Sci Adv. 2019 Jul 31;5(7):eaav6313. doi: 10.1126/sciadv.aav6313 (PMC6669016; doi:10.1126/sciadv.aav6313)
Supplement: Download PDF [file aav6313_SM.pdf]

Science Advances
AAAS

advances.sciencemag.org/cgi/content/full/5/7/eaav6313/DC1

Supplementary Materials for

Treating ischemia via recruitment of antigen-specific T cells

Brian J. Kwee, Bo Ri Seo, Alexander J. Najibi, Aileen W. Li, Ting-Yu Shih, Des White, David J. Mooney*

*Corresponding author. Email: mooneyd@seas.harvard.edu

Published 31 July 2019, *Sci. Adv.* **5**, eaav6313 (2019)
DOI: 10.1126/sciadv.aav6313

This PDF file includes:

Fig. S1. Scaffold implantation on ischemic ligation.
Fig. S2. Representative FACS gating strategy for quantifying percentage and number of different immune cells.
Fig. S3. Distribution of $CD4^+$ T cells recruited to scaffold and upper leg muscles.
Fig. S4. Recruitment of $CD4^+$ T cells in OT-II mice.
Fig. S5. OVA/ALUM vaccination enhances IL-5–producing OVA-specific $CD4^+$ T cells in BALB/c mice.
Fig. S6. Concentration of T_H2 $CD4^+$ T cells in ischemic hindlimb muscle.
Fig. S7. Images of wells from IL-5 ELISPOT assay, measuring IL-5–secreting cells from cells isolated from ischemic thighs 4 days after ischemic ligation.
Fig. S8. Images of wells from IL-5 ELISPOT assay, measuring IL-5–secreting cells from cells isolated from ischemic thighs 7 days after ischemic ligation.
Fig S9. Concentration of T_H1/T_H2 cytokines secreted by OVA-stimulated cells in ischemic hindlimb muscle.
Fig. S10. Distribution of eosinophils recruited to scaffold and upper leg muscles.
Fig. S11. Presence of α-SMA^+ blood vessels in tissue adjacent to scaffold.
Fig. S12. Antigen-releasing scaffolds enhance blood perfusion recovery following ischemic injury in an antigen-specific manner.
Fig. S13. Blood perfusion recovery in vaccinated mice with OVA-releasing scaffolds depends on the presence of $CD4^+$ T cells.
Fig. S14. Characterization of types of muscle fibers in histological sections of ischemic lower leg muscles.

Supplementary Materials

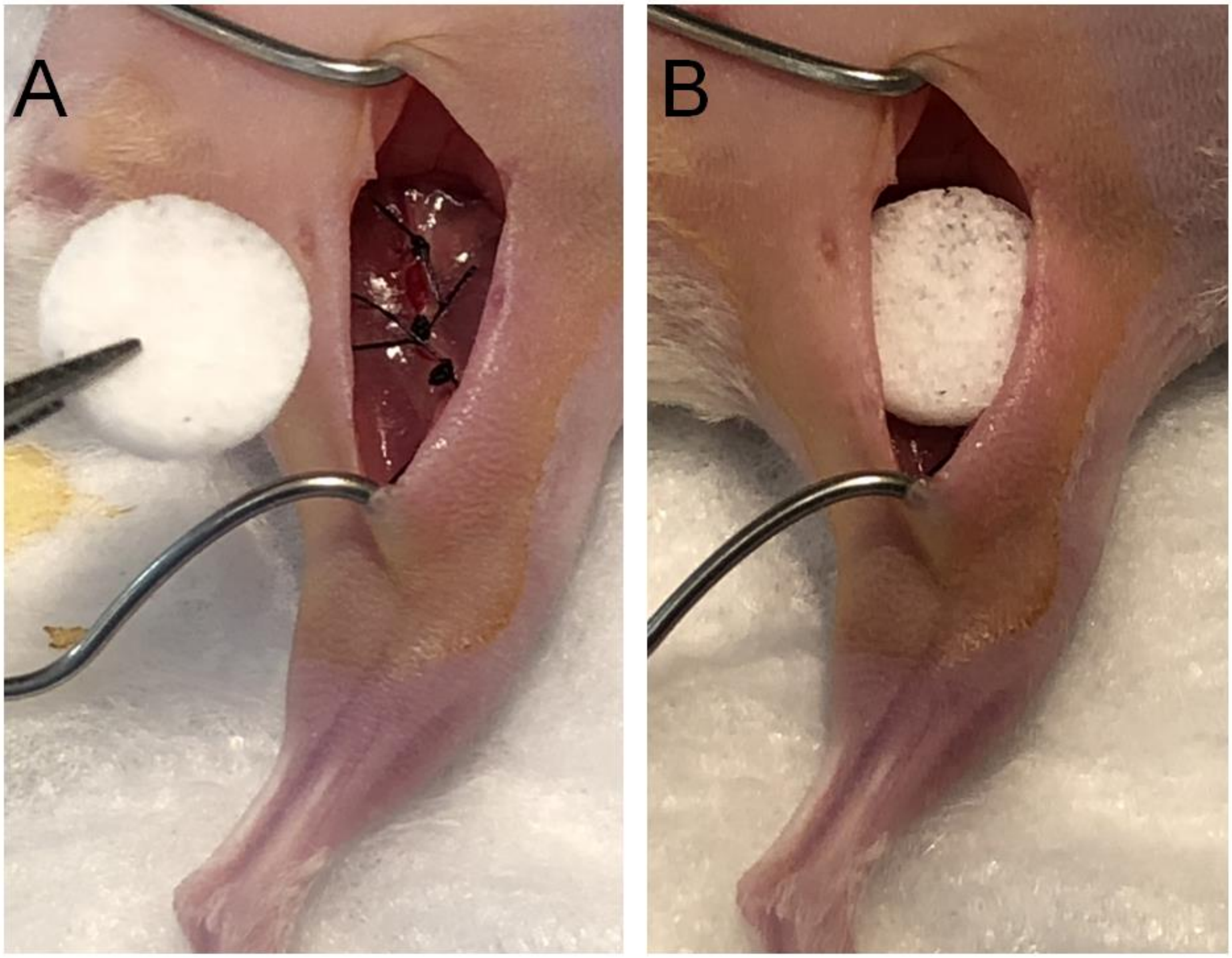

Fig. S1. Scaffold implantation on ischemic ligation. Intraoperative image of ischemic ligation surgery showing (**A**) PLG scaffold (8 mm in diameter) size relative to sites of ligation on external iliac and femoral artery, and (**B**) implantation site of PLG scaffold on ischemic ligation sites. (photo credit: Brian Kwee, Harvard University)

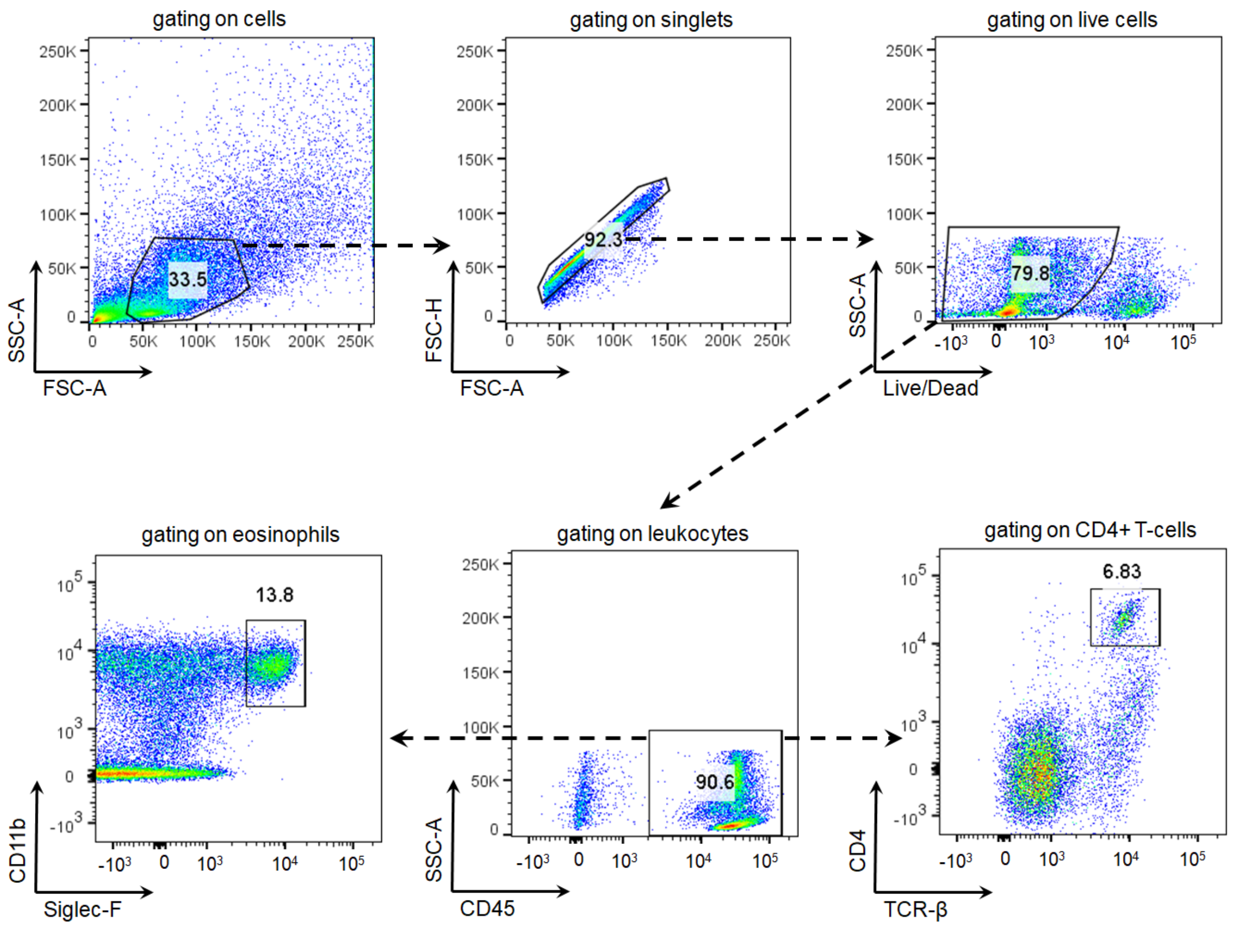

Fig. S2. Representative FACS gating strategy for quantifying percentage and number of different immune cells. Cells were first gated on a SSC-A vs. FSC-A plot to exclude debris. Single cells were then gated based on a FSC-H vs. FSC-A plot. Live cells were then gated using fixable live/dead stain. The percentage of leukocytes was then determined based on CD45 staining. The percentage of specific immune cells, such as TCR-β+/CD4+ helper T-cells or CD11b+/Siglec-F+ eosinophils, were then gated.

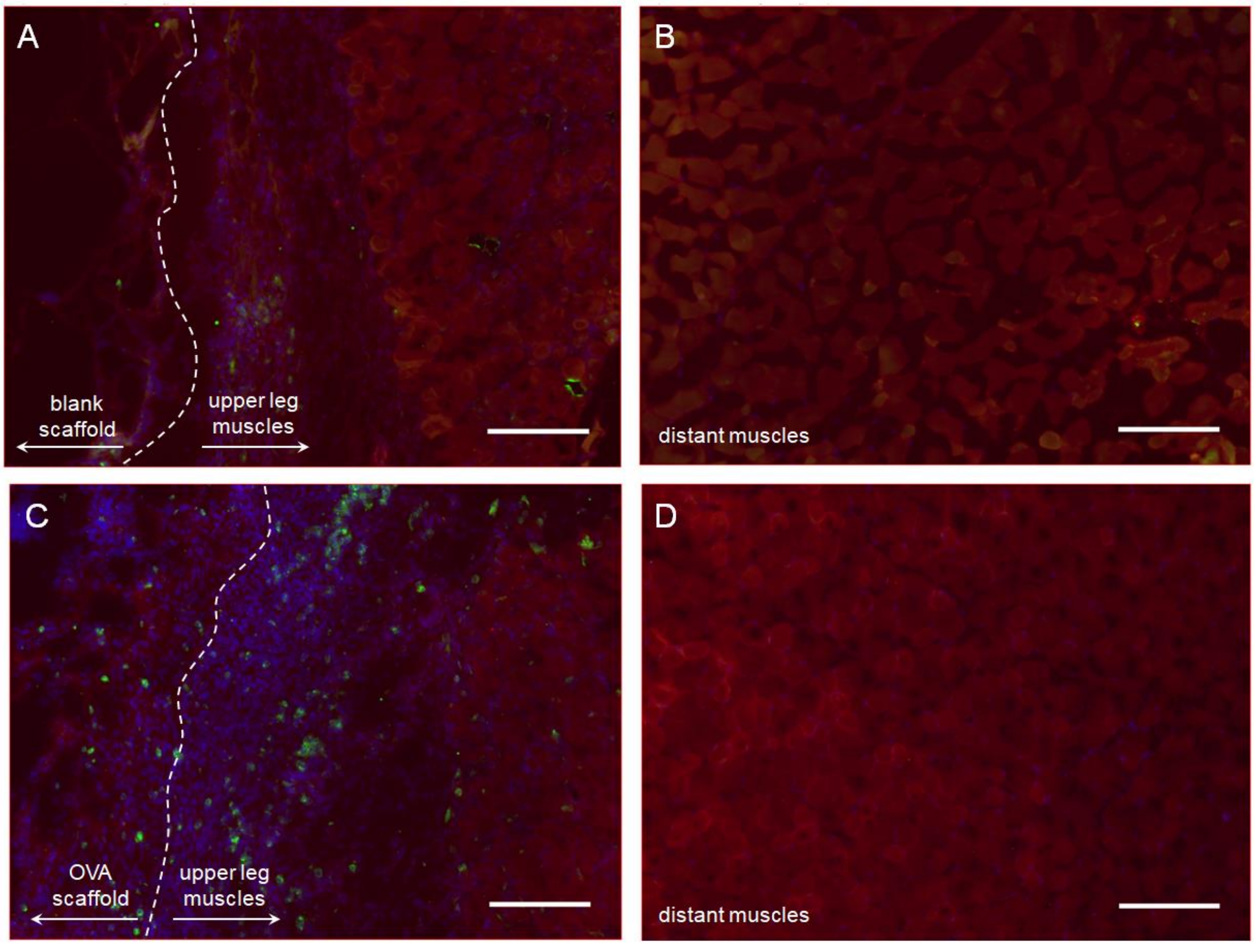

Fig. S3. Distribution of CD4$^+$ T cells recruited to scaffold and upper leg muscles. Representative histology images showing distribution of CD4$^+$ T-cells recruited to the boundary between the scaffold and upper leg muscles, and skeletal muscle distant from the scaffold in OT-II mice treated with (**A**,**B**) blank scaffold and (**C**,**D**) OVA-containing scaffold. Analysis at 7 days post-ischemic ligation and treatment. White dotted line indicates boundary between scaffold and upper leg muscles (green = CD4+ cells, blue = DAPI, red = autofluoresence of muscle in GFP channel). Scale bar = 200 µm.

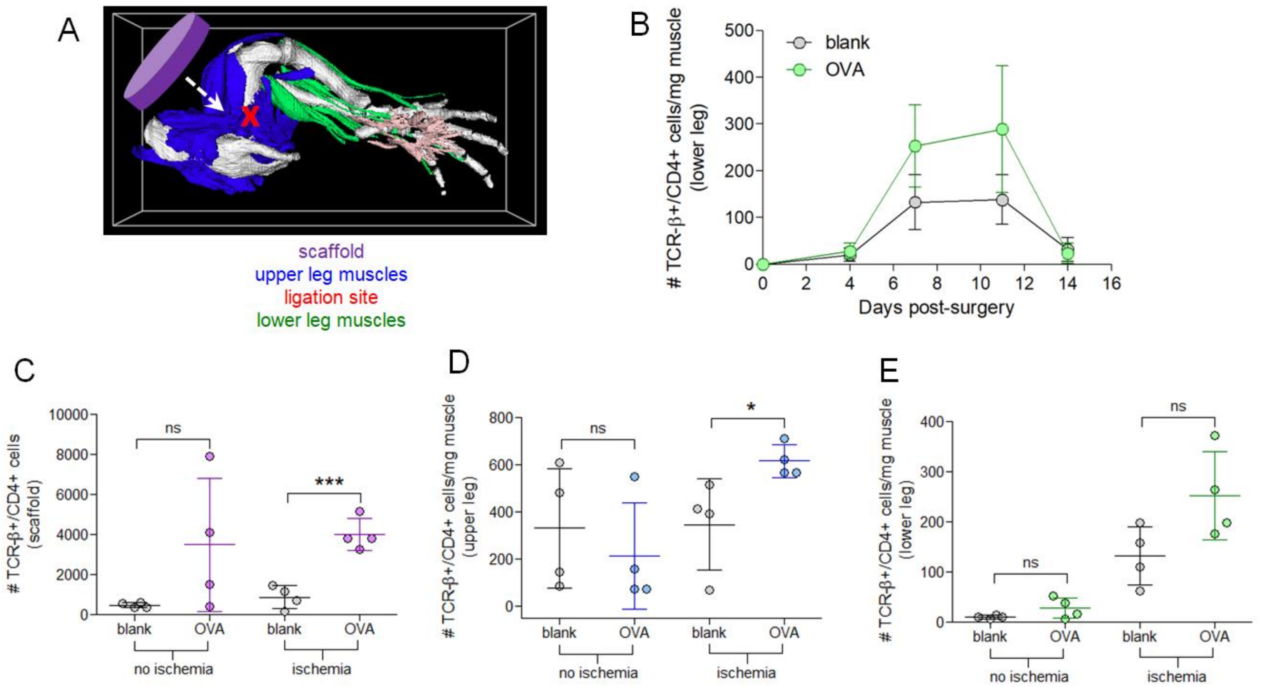

Fig. S4. Recruitment of CD4$^+$ T cells in OT-II mice. (**A**) Diagram of scaffold implantation (purple) relative to ischemic upper leg muscles (blue), ligation site (red), and lower leg muscles (green). White arrow indicates location of placement of scaffold in diagram. (**B**) Number of CD4$^+$ T-cells (TCR-β+/CD4+ cells) in the lower leg muscle per mg of tissue over time (n = 4 mice). (**C-E**) Number of CD4$^+$ T-cells (TCR-β+/CD4+ cells) in the (C) scaffold, (D) upper leg muscles, and (E) lower leg muscles per mg of tissue at day 7 post scaffold implantation with and without ischemia (n = 4 mice). Data represented as mean ± S.D. Significance is denoted by *P≤0.05, ***P≤0.001 with a two-tailed student's *t*-test with or without Welch's correction where applicable.

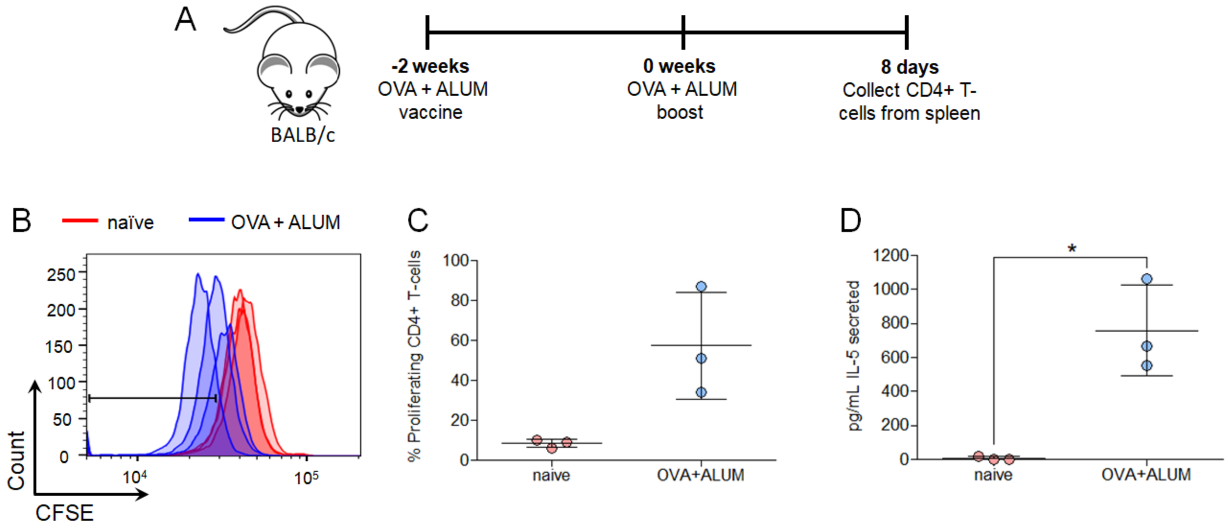

Fig. S5. OVA/ALUM vaccination enhances IL-5–producing OVA-specific CD4$^+$ T cells in BALB/c mice. (**A**) Experimental set up with (left) BALB/c mice, undergoing (right) timeline of procedures including vaccination and boost with OVA/ALUM and collection of CD4$^+$ T-cells from spleen. (**B**) Representative FACS plots of CD4$^+$ T-cells from naive or OVA/ALUM vaccinated mice stained with CFSE, cultured in the presence BMDCs presenting OVA (n = 3 mice). (**C**,**D**) Quantification of (C) percent proliferating CD4$^+$ T-cells and (D) concentration of IL-5 secreted by CD4$^+$ T-cells from treated mice (n = 3 mice). Data represented as mean ± S.D. Significance is denoted by *P≤0.05 by a two-tailed student's *t*-test with or without Welch's correction where applicable.

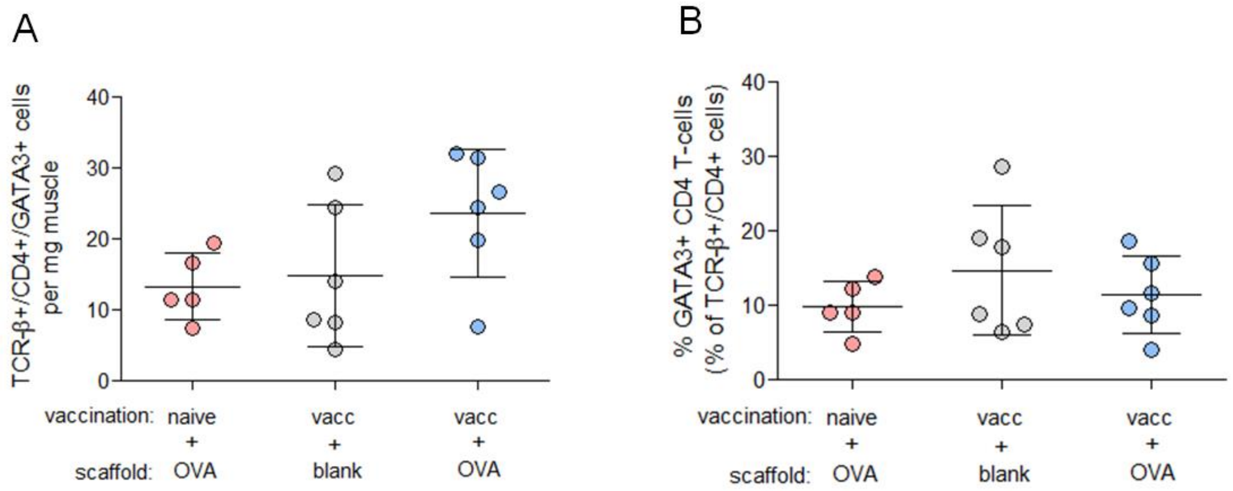

Fig. S6. Concentration of T_H2 CD4$^+$ T cells in ischemic hindlimb muscle. In these figures, 'naive+OVA' denotes mice receiving no vaccination and implanted with an OVA-containing scaffold, 'vacc+blank' denotes mice receiving vaccination with OVA/ALUM and a blank scaffold, and 'vacc+OVA' denotes mice receiving vaccination with OVA/ALUM and an OVA-containing scaffold. (**A**) Number of total Th2 CD4$^+$ T-cells (TCR-β+/CD4+/GATA3+ cells) per mg of tissue in ischemic upper leg at day 4 post-ischemic ligation in various treated groups. (**B**) Percent Th2 T-cells (of total CD4$^+$ T-cells) in ischemic upper leg at day 4 post-ischemic ligation in various treated groups. n = 5 mice for 'naive+OVA', n = 6 mice for 'vacc+blank', and n = 6 mice for 'vacc+OVA'. Data represented as mean ± S.D.

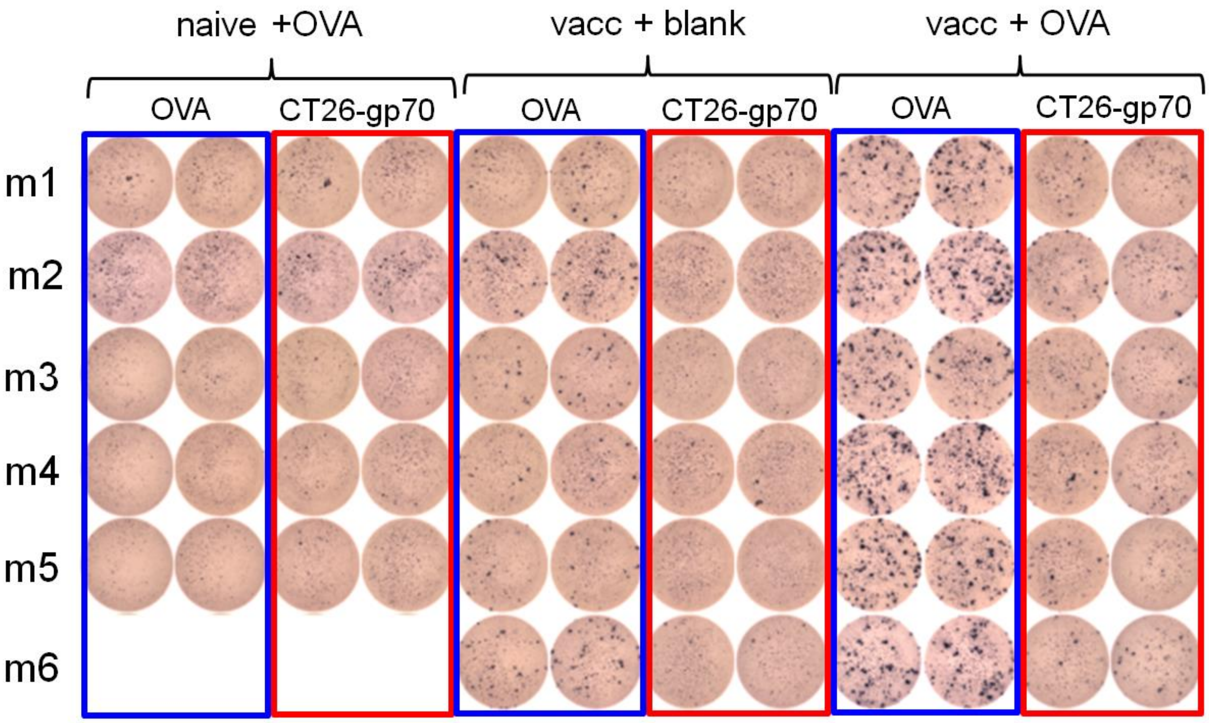

Fig. S7. Images of wells from IL-5 ELISPOT assay, measuring IL-5–secreting cells from cells isolated from ischemic thighs 4 days after ischemic ligation. In this figure, 'naive+OVA' denotes mice receiving no vaccination and implanted with an OVA-containing scaffold, 'vacc+blank' denotes mice receiving vaccination with OVA/ALUM and a blank scaffold, and 'vacc+OVA' denotes mice receiving vaccination with OVA/ALUM and an OVA-containing scaffold. Cells derived from ischemic muscle were cultured in the presence of 10 μg/mL OVA or 10 μg/mL CT26-gp70 with splenocytes from naive mice. Each row represents cells from a different mouse, tested with each peptide in duplicate. n = 5 mice for 'naive+OVA', n = 6 mice for 'vacc+blank', and n = 6 mice for 'vacc+OVA'.

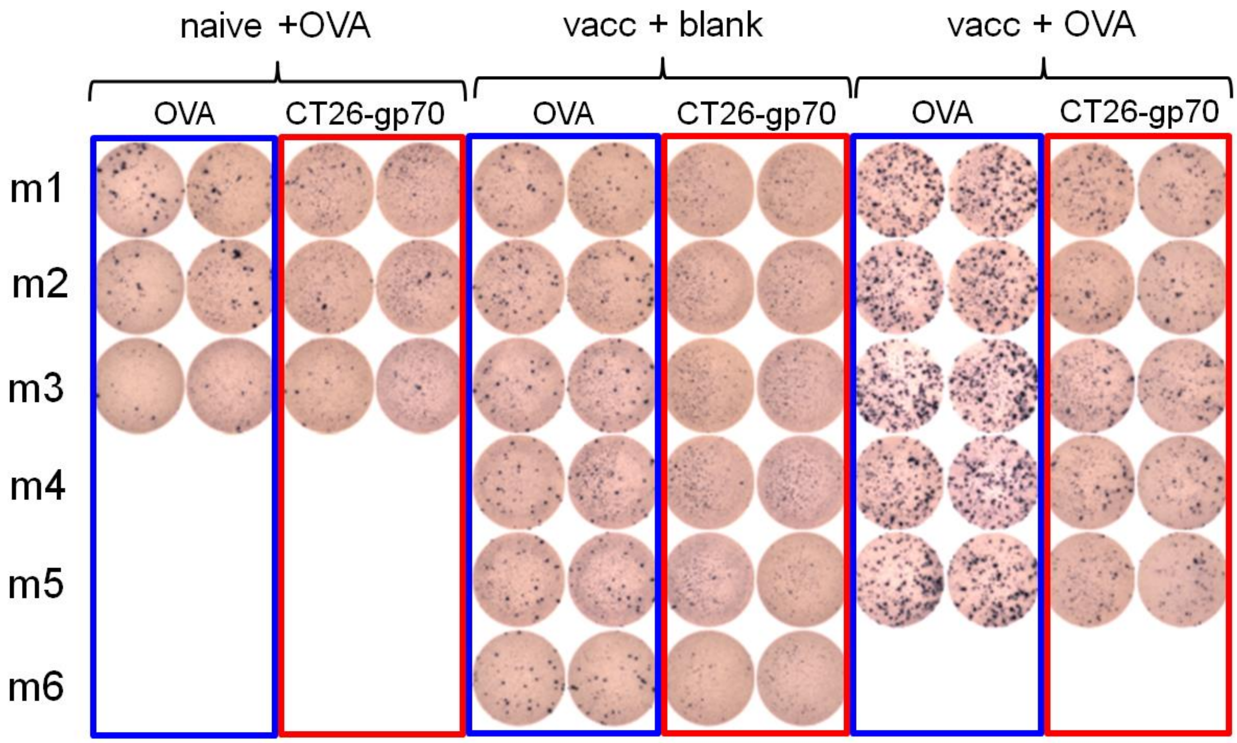

Fig. S8. Images of wells from IL-5 ELISPOT assay, measuring IL-5–secreting cells from cells isolated from ischemic thighs 7 days after ischemic ligation. In this figure, 'naive+OVA' denotes mice receiving no vaccination and implanted with an OVA-containing scaffold, 'vacc+blank' denotes mice receiving vaccination with OVA/ALUM and a blank scaffold, and 'vacc+OVA' denotes mice receiving vaccination with OVA/ALUM and an OVA-containing scaffold. Cells derived from ischemic muscle cultured in the presence of 10 µg/mL OVA or 10 µg/mL CT26-gp70 with splenocytes from naive mice. Each row represents cells from a different mouse, tested with each peptide in duplicate. n = 3 mice for 'naive+OVA', n = 6 mice for 'vacc+blank', and n = 5 mice for 'vacc+OVA'.

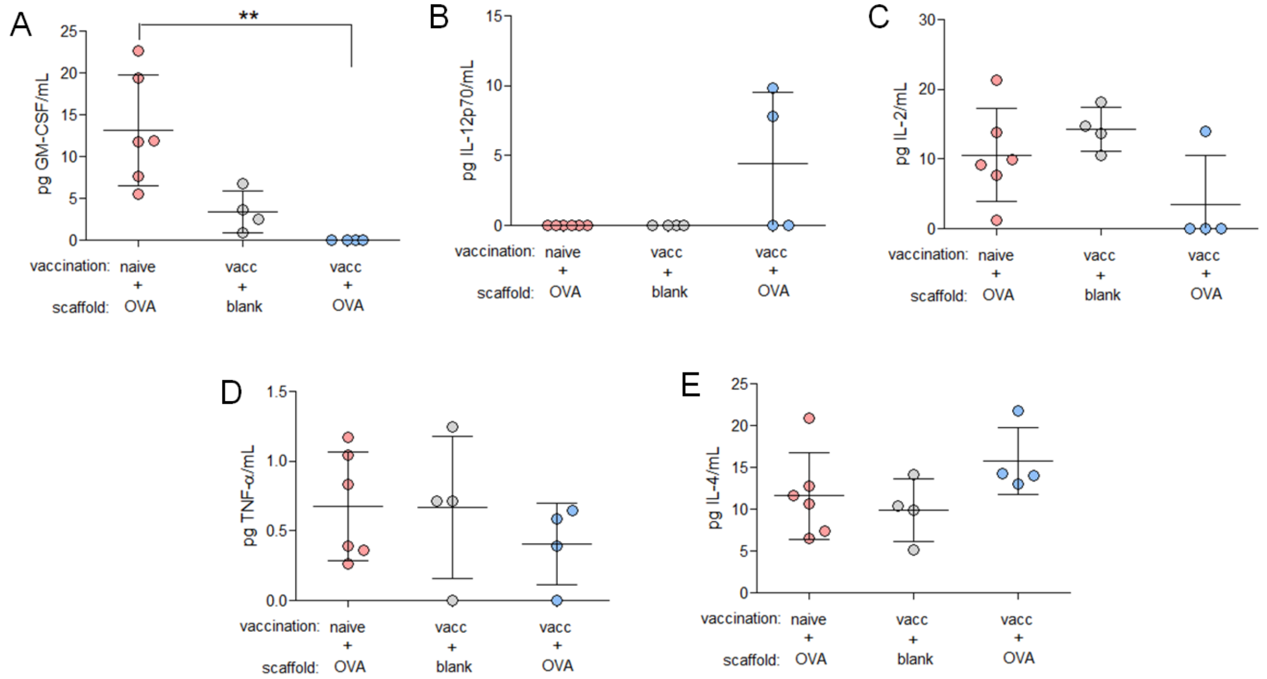

Fig S9. Concentration of T_H1/T_H2 cytokines secreted by OVA-stimulated cells in ischemic hindlimb muscle. In these figures, 'naive+OVA' denotes mice receiving no vaccination and implanted with an OVA-containing scaffold, 'vacc+blank' denotes mice receiving vaccination with OVA/ALUM and a blank scaffold, and 'vacc+OVA' denotes mice receiving vaccination with OVA/ALUM and an OVA-containing scaffold. Concentrations of (**A**) GM-CSF, (**B**) IL-12p70, (**C**) IL-2, (**D**) TNF-α, and (**E**) IL-4 produced from OVA-stimulated cells isolated from upper leg muscles 7 days post-ischemic ligation. n = 6 mice for 'naive+OVA', n = 4 mice for 'vacc+blank', and n = 4 mice for 'vacc+OVA'. Data represented as mean ± S.D. Significance is denoted by $^{**}P \leq 0.01$ by one-way ANOVA with Bonferroni's post-hoc test.

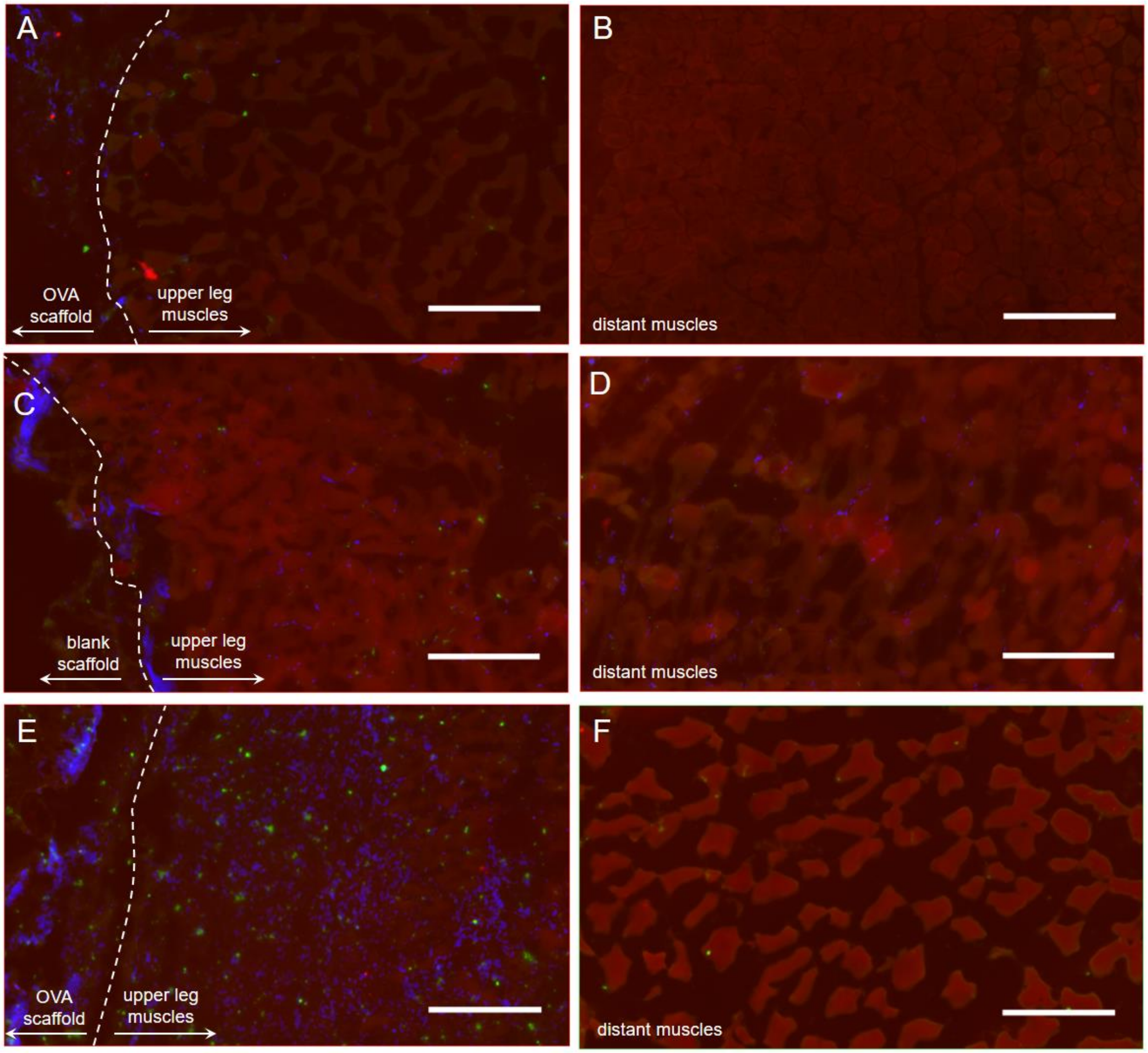

Fig. S10. Distribution of eosinophils recruited to scaffold and upper leg muscles. Representative histology images showing distribution of eosinophils recruited to the boundary between the scaffold and upper leg muscles, and skeletal muscle distant from the scaffold in (**A**,**B**) mice receiving no vaccination and implanted with an OVA-containing scaffold, (**C**,**D**) mice receiving vaccination with OVA/ALUM and a blank scaffold, and (**E**,**F**) mice receiving vaccination with OVA/ALUM and an OVA-containing scaffold. Analysis at 7 days post-ischemic ligation and treatment. White dotted line indicates boundary between scaffold and upper leg muscles (green = Siglec-F+ cells, blue = DAPI, red = autofluoresence of muscle in GFP channel). Scale bar = 200 µm.

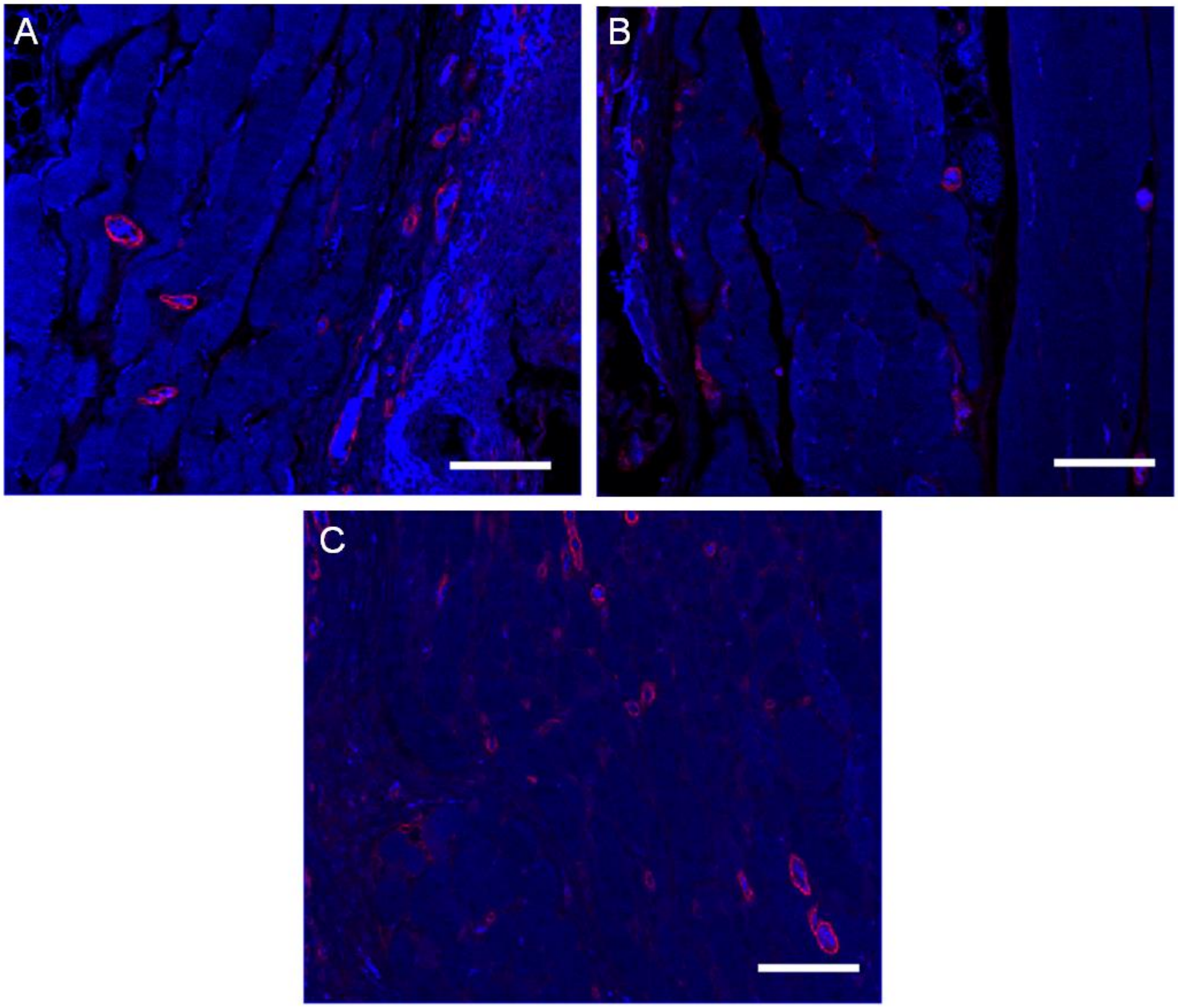

Fig. S11. Presence of α-SMA^{+} blood vessels in tissue adjacent to scaffold. Representative histology sections of α-SMA+ blood vessels in ischemic tissue adjacent to scaffold in upper leg muscles at 14 days post-ischemic ligation in (**A**) mice receiving no vaccination and implanted with an OVA-containing scaffold, (**B**) mice receiving vaccination with OVA/ALUM and a blank scaffold, and (**C**) mice receiving vaccination with OVA/ALUM and an OVA-containing scaffold (red = α-SMA, blue = muscle autofluoresense in GFP channel). Scale bar = 100 µm.

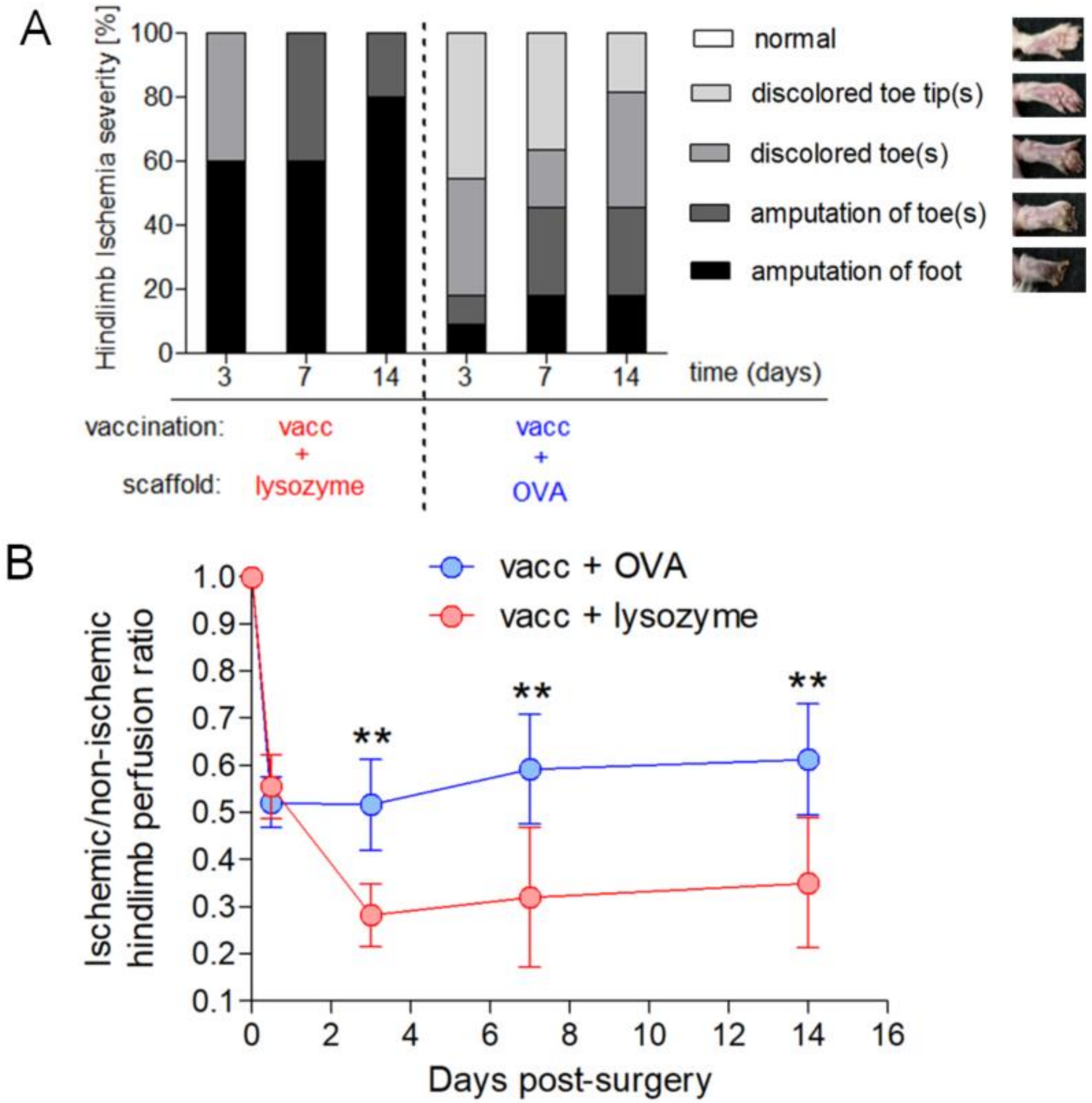

Fig. S12. Antigen-releasing scaffolds enhance blood perfusion recovery following ischemic injury in an antigen-specific manner. In these figures, 'vacc+OVA' denotes mice receiving vaccination with OVA/ALUM and an OVA-containing scaffold and 'vacc+lysozyme' denotes mice receiving vaccination with OVA/ALUM and a lysozyme-containing scaffold. (**A**) Ischemic hindlimbs with various treatment groups were visually examined to determine the severity of hindlimb ischemia at 3, 7, and 14 days post-ischemic ligation (n = 11 mice for 'vacc+OVA' and n = 5 mice for 'vacc+lysozyme'). (**B**) Quantification of ischemic to non-ischemic blood perfusion ratio in various treated mice over the course of 14 days post-ischemic ligation (n = 10-11 mice for 'vacc+OVA' and n = 3-5 mice for 'vacc+lysozyme'). Data represented as mean ± S.D. Significance is denoted by **$P \leq 0.01$ by a two-tailed student's *t*-test with or without Welch's correction where applicable. (photo credit: Brian Kwee, Harvard University)

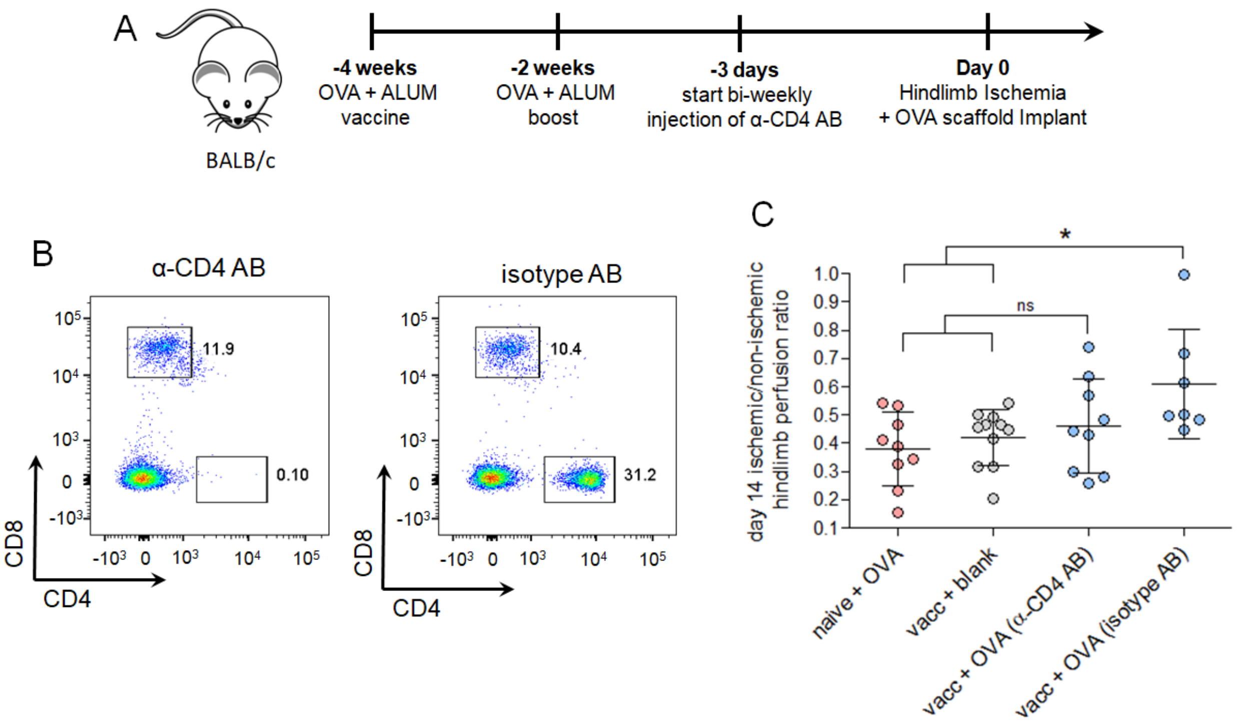

Fig. S13. Blood perfusion recovery in vaccinated mice with OVA-releasing scaffolds depends on the presence of $CD4^+$ T cells. In these figures, 'naive+OVA' denotes mice receiving no vaccination and implanted with an OVA-containing scaffold, 'vacc+blank' denotes mice receiving vaccination with OVA/ALUM and a blank scaffold, and 'vacc+OVA' denotes mice receiving vaccination with OVA/ALUM and an OVA-containing scaffold. (**A**) Experimental set up with (left) BALB/c mice undergoing (right) timeline of procedures involving vaccination and boost with OVA/ALUM, biweekly injections of neutralizing anti-CD4 antibody, and hindlimb ischemia ligation and scaffold implantation. (**C**) Representative flow cytometry analysis of blood T-cells six days after treatment with anti-CD4 monoclonal antibody or an isotype antibody. (**D**) Quantification of ischemic to non-ischemic blood perfusion ratio of 'naive + OVA' treated mice (n = 9 mice), 'vacc + blank' treated mice (n = 11 mice), 'vacc+OVA' treated mice injected with anti-CD4 antibody (n = 9 mice), and 'vacc+OVA' treated mice injected with isotype antibody (n = 7 mice) 14 days post-ischemic ligation. Data represented as mean ± S.D. Significance is denoted by *$P \leq 0.05$ by one-way ANOVA with Bonferroni's post-hoc test.

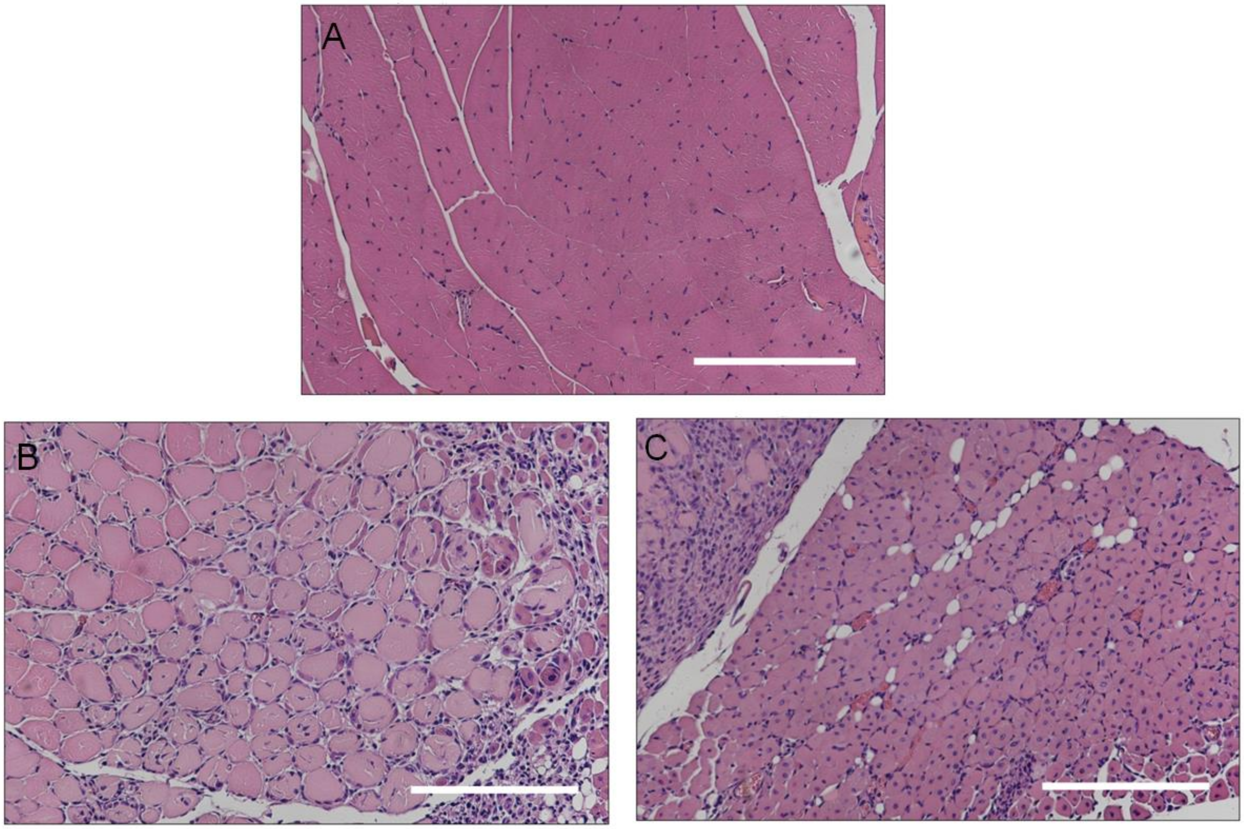

Fig. S14. Characterization of types of muscle fibers in histological sections of ischemic lower leg muscles. Representative H&E stained histology images of (**A**) normal muscle fibers, (**B**) muscles fibers with ischemic coagulation necrosis (fragmented muscle fibers that are paler in color compared to normal muscle fibers), and (**C**) regenerating muscle fibers (centrally nucleated muscle fibers). Scale bar = 200 μm.
